# Exploration of Spirocyclic Derivatives of Ciprofloxacin as Antibacterial Agents

**DOI:** 10.3390/molecules27154864

**Published:** 2022-07-29

**Authors:** Alexei Lukin, Mikhail Chudinov, Tatiana Vedekhina, Elizaveta Rogacheva, Lyudmila Kraeva, Olga Bakulina, Mikhail Krasavin

**Affiliations:** 1 Lomonosov Institute of Fine Chemical Technologies, MIREA—Russian Technological University, 119454 Moscow, Russia; alex-look@yandex.ru (A.L.); mikle@irims.ru (M.C.); 2 Federal Research and Clinical Center of Physico-Chemical Medicine, 1a Malaya Pirogovskaya Street, 119435 Moscow, Russia; neglect1@yandex.ru; 3 Pasteur Institute of Epidemiology and Microbiology, 14 Mira Street, 197101 Saint Petersburg, Russia; elizvla@yandex.ru (E.R.); lykraeva@yandex.ru (L.K.); 4 Saint Petersburg State University, 26 Universitetskii Prospect, 198504 Peterhof, Russia; bakulina-o.y@yandex.ru; 5 Immanuel Kant Baltic Federal University, 236041 Kaliningrad, Russia

**Keywords:** antibacterial, ciprofloxacin, aromatic nucleophilic substitution, spirocyclic, piperidines

## Abstract

The previously reported as well as newly synthesized derivatives of the 1-oxa-9-azaspiro[5.5]undecane were employed in the synthesis of thirty-six derivatives of ciprofloxacin using commercially available 7-chloro-1-cyclopropyl-6-fluoro-4-oxo-1,4-dihydroquinoline-3-carboxylic acid and the literature protocol involving the preparation of boron chelate complex to facilitate nucleophilic aromatic substitution. All new fluoroquinolone derivatives were tested against two gram-positive as well as three gram-negative strains of bacteria. With the activity spectrum of the new derivatives being substantially narrower than that of ciprofloxacin, compounds were distinctly active against two of the five strains: gram-negative *Acinetobacter baumannii* 987^®^ and gram-positive *Bacillus cereus* 138^®^. Towards these two strains, a large group of compounds displayed equal or higher potency than ciprofloxacin.

## 1. Introduction

Spirocycles represent an emerging privileged structural class for drug design [1]. Not only are spirocyclic motifs omnipresent in the natural product realm [2]; they are also employed, with increasing frequency, in drug candidate development [3]. The latter observation most likely attests to the widespread recognition of the unique structural properties offered by spirocyclic frameworks. These include, though are not limited to, the pronounced three-dimensional character of spirocycles [4], the well-defined spatial projection of peripheral appendages off the spirocyclic scaffold [5], the inherent high degree of saturation (defined as F_sp3_ or fraction of sp^3^-hybridized heavy atoms) [6] and the presence of multiple stereocenters. Certainly, all these aspects do not necessarily facilitate the still daunting task of the finding of a new, biologically active lead molecule acting via a specific biological target. However, if such a molecule is identified around a spirocyclic scaffold or with the use of spirocyclic periphery motifs, optimizing it into potent, selective and overall developable [7] drug candidate is an undertaking less prone to failure due to ligand promiscuity, undesired metabolism and pharmacokinetics or overall unfavorable physicochemical profile [8]. In other words, the use of spirocycles in drug design front-loads many important aspects of drug discovery and development which are traditionally worried about at more advanced stages of the process [9].

**Scheme 1:**
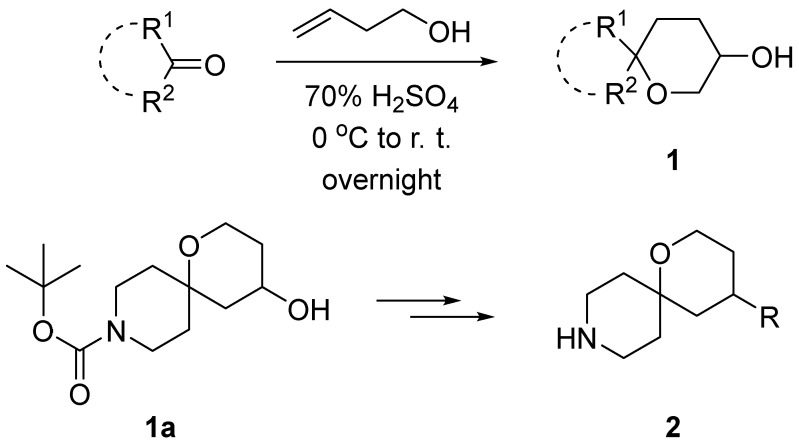
Synthesis of spirocyclic building blocks **1** via the Prins cyclization and subsequent diversification of spirocyclic piperidine **1a**.

**Figure 1:**
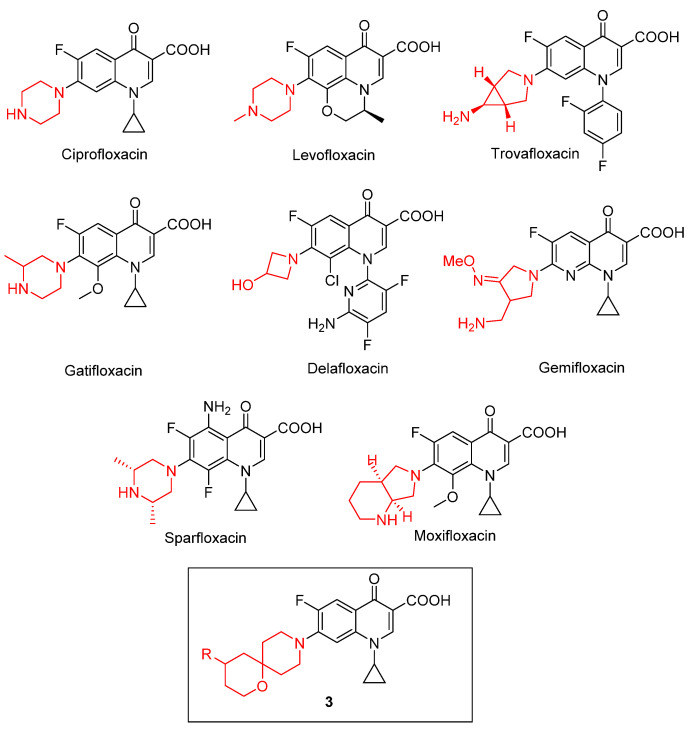
Structures of exemplary approved fluoroquinolone antibacterials as well as fluoroquinolone chemotype **3** explored in this work.

In 2016, we developed a facile synthetic entry into spirocyclic building blocks **1** via the sulfuric-acid-promoted Prins cyclization of cyclic ketones with homoallylic alcohol [10]. Besides the general utility of these building blocks for drug discovery, one particular compound, *N*-Boc-protected spirocyclic piperidine **1a,** was synthesized on a multigram scale and was envisioned as a starting material for a larger cluster of diversely substituted spirocyclic piperidines **2** for various medicinal applications (Scheme 1). In particular, many of these spirocyclic building blocks were successfully employed in the design of free fatty acid receptor 1 (GPR40) agonists for the treatment of type 2 diabetes mellitus. The synthesis was based on the conjugation of these building blocks to a fatty-acid-mimicking carboxylic acid warhead [11,12,13]. More recently, we followed a similar strategy in antibacterial area and attached these spirocycles to the pharmacophoric 5-nitrofuroyl moiety, obtaining a series of non-toxic nitrofurans that turned out to be efficacious in vitro against multidrug-resistant *Mycobacterium tuberculosis* [14]. Inspired by the propensity of spirocycles **2** to deliver the chemotypes of desired biological profiles depending on the nature of the pharmacophoric element, we continued to further exploit the privileged [15] character of this versatile medicinal chemistry toolkit. Employing compounds **2** in the design and synthesis of fluoroquinolone antibacterials such as ciprofloxacin became our next focal point. It is well known that variation of cyclic secondary amine appendages at position 7 of the quinolone ring (alone or in combination with other alterations of the core as well as the periphery) of this potent class of antibiotics has a strong bearing on the antimicrobial profile and has delivered numerous efficacious, broad-spectrum antibiotics [16]. Hence, using the already-amassed arsenal of building blocks **2** as well as several newly prepared derivatives, we aspired to synthesize and evaluate the antimicrobial profile of fluoroquinolones **3** (Figure 1). Herein, we report on the results obtained in the course of realizing this strategy.

## 2. Results

**Figure 2:**
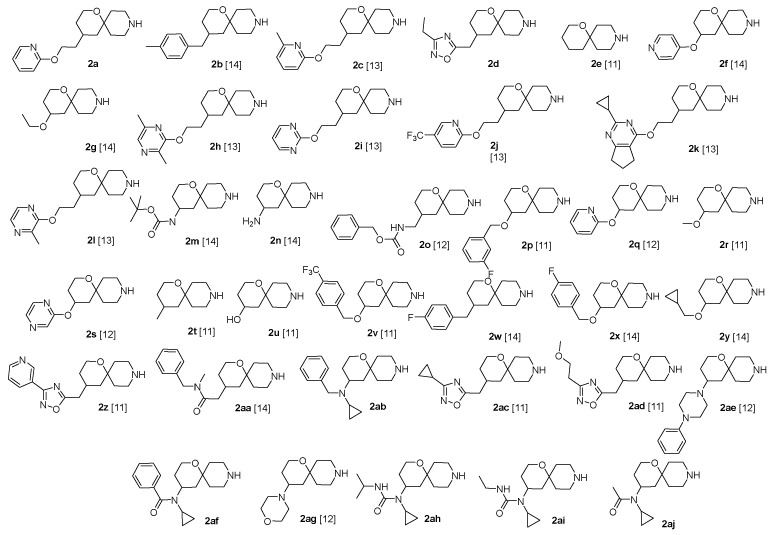
Spirocyclic piperidines **2a**–**aj** employed in this study [11,12,13,14].

**Scheme 2:**
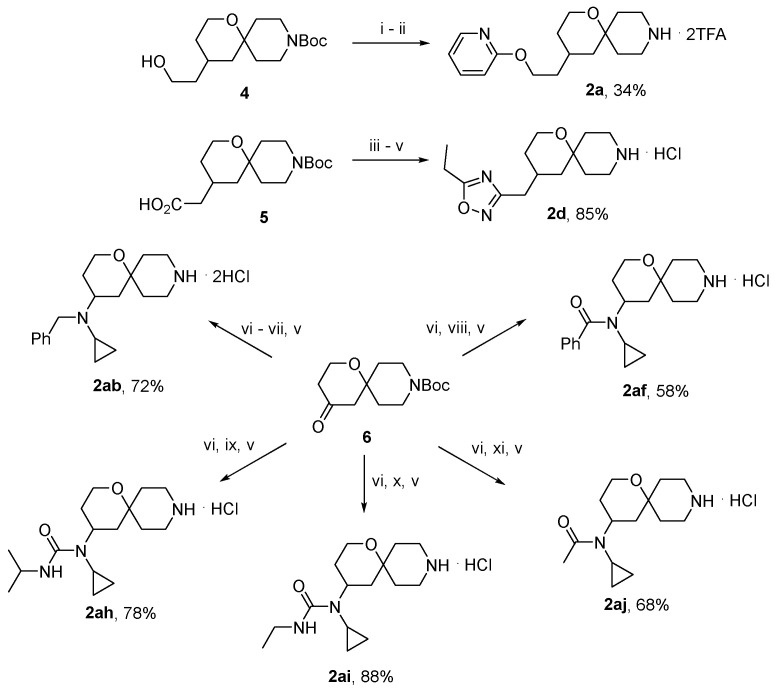
Synthesis of novel spirocyclic piperidines **2a**, **2d**, **2ab**, **2af**, **2ah**, **2ai** and **2aj**. *Reagents and conditions:* i. NaH, DMF, 2-chloropyridine, 0 °C → r. t., 16 h; ii. TFA, DCM, 0 °C, 6 h; iii. *i*BuOCOCl, *N*-methylmorpholine, THF, −30 → −5 °C; iv. EtC(=NOH)NH_2_; v. 4M HCl, 1,4-dioxane, 0 °C → r. t., 6 h; vi. cyclopropylNH_2_, NaBH(OAc)_3_, DCM, r. t., 18 h; vii. PhCHO, NaBH(OAc)_3_, DCM, r. t., 18 h; viii. PhCOCl, Et_3_N, DCM, r. t., 6 h; ix. *i*PrNCO, 1,4-dioxane, r. t., 6 h; x. EtNCO, 1,4-dioxane, r. t., 6 h; xi. AcCl, Et_3_N, DCM, r. t., 6 h.

For the synthesis of the library of novel fluoroquinolone analogs of ciprofloxacin, 36 spirocyclic piperidines **2a**–**aj** were selected (Figure 2). The synthesis of the majority of these compounds had been reported previously [11,12,13,14] while seven building blocks (**2a**, **2d**, **2ab**, **2af**, **2ah**, **2ai** and **2aj**) were synthesized from the earlier reported starting materials **4** [13], **5** [11] and **6** [11] as shown in Scheme 2.

**Scheme 3:**
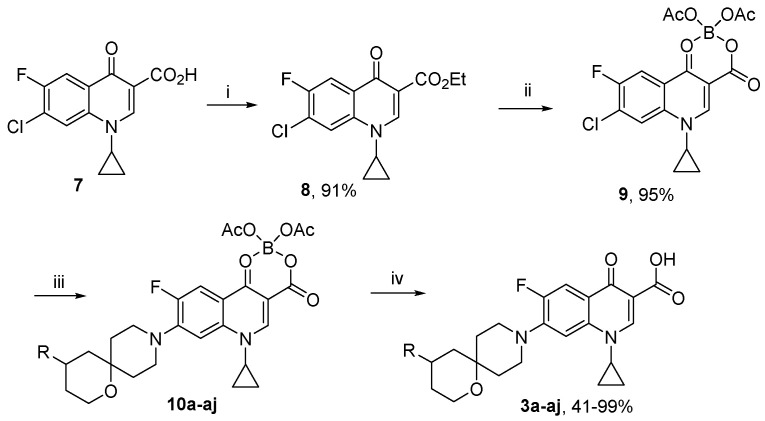
Synthesis of target spirocyclic-periphery fluoroquinolones **3a**–**aj**. *Reagents and conditions:* i. K_2_CO_3_, EtBr, DMF, r. t., 10 h; ii. H_3_BO_3_, Ac_2_O, ZnCl_2_, r. t. 60 °C; iii. **2a**–**aj**, Et_3_N, MeCN, 10 h, 60 °C; iv. 2% aq. NaOH.

The synthesis of target fluoroquinolones **3a**–**aj** commenced with commercially available 7-chloro-1-cyclopropyl-6-fluoro-4-oxo-1,4-dihydroquinoline-3-carboxylic acid (**7**) which was esterified to give ester **8**. The latter was converted to boron chelation complex **9** using the published protocols [17,18,19]. In the latter, the chlorine in position 7 is particularly activated towards the nucleophilic aromatic substitution. The latter was brought about by heating compound **9** with spirocyclic piperidines **2a**–**aj** at 60 °C in the presence of triethylamine. The boron chelation complex [20] was removed by exposing intermediates **10a**–**aj** to a 2% aqueous sodium hydroxide solution. As a result, fluoroquinolones **3a**–**aj** were obtained in yields from moderate to nearly quantitative (Scheme 3).

Having synthesized spirocyclic derivatives of fluoroquinolone ciprofloxacin **3a**–**aj**, we proceeded to evaluate their antibacterial profile against two gram-positive (*Staphylococcus aureus* ATCC 25923 and *Bacillus cereus* 138^®^) as well as three gram-negative (*Klebsiella pneumoniae* 1062^®^, *Acinetobacter baumannii* 987^®^ and *Pseudomonas aeruginosa* 7292/5^®^) strains of bacteria (^®^ indicates clinical isolate strains from the Pasteur Institute’s own collection of bacterial strains). Ciprofloxacin was used in these assays as a positive control.

As is evident from the data presented in Table 1, the 36 compounds displayed varying degrees of activity against different strains, in contrast to ciprofloxacin which acted as a broad-spectrum antibiotic across the panel of all five strains. Clearly, the new set of fluoroquinolones possesses a narrower spectrum of activity compared to ciprofloxacin. However, the propensity of the newly synthesized set of fluoroquinolones to kill gram-negative *Acinetobacter baumannii* 987^®^ bacteria is evident. Indeed, eight compounds (**3d**, **3f**, **3j**–**k**, **3q**, **3r**, **3u**, **3ae**) were equipotent to ciprofloxacin against this strain while fourteen compounds (**3b**, **3e**, **3g**, **3l**, **3n**–**p**, **3v**–**w**, **3z**, **3aa**–**ab**, **3ad**, **3af**) had an even lower minimum inhibitory concentration (MIC) than ciprofloxacin. The other bacterium that was significantly affected by the best compounds in the set was gram-positive *Bacillus cereus* 138^®^. Out of 36 compounds tested, nine (**3f**–**g**, **3l**, **3n**, **3r**, **3u**, **3ac**–**ad**, **3ag**) had the same activity towards this strain as ciprofloxacin. In contrast to the latter, none of the compounds (except for weakly active **3d**) displayed activity towards gram-negative *Pseudomonas aeruginosa* 7292/5^®^. Additionally, the new spirocyclic derivatives were only weakly active towards gram-positive *Staphylococcus aureus* ATCC 25923 and gram-negative *Klebsiella pneumoniae* 1062^®^.

Overall, the antibacterial activity of the fluoroquinolones studied turned out to be rather sensitive to the nature of the molecular periphery. Indeed, while some of the 2-(azin-2-yl)oxyethyl-substituted compounds (**3a**, **3c**, **3h**) were virtually inactive (except for some weak activity against *Staphylococcus aureus* ATCC 25923), closely related analogs **3i**–**l** were substantially more active, with **3j**–**l** being equipotent or even more potent towards *Acinetobacter baumannii* 987^®^ compared to ciprofloxacin. For the high potency towards the latter strain, no periphery around the 1-oxa-9-azaspiro[5.5]undecane spirocycles (**3e**) or small substituents such as ethoxy (**3g**), amino (**3l**), methoxy (**3r**) or hydroxy (**3u**) appears to suffice. At the same time, rather elaborate periphery groups (**3o**–**p**, **3v**–**w**, **3z**, **3aa**–**3ab**, **3ad**, **3af**) also resulted in high activity against *Acinetobacter baumannii* 987^®^. An interesting observation could be made about the basic character of peripheral R groups in compounds **3**. The spectrum of bactericidal activity appears to be broader for protonatable (*cf*. **3f**, **3n**, **3ag**) or hydrogen-bond-donating (**3u**) substituents. With respect to broader, ciprofloxacin-like (albeit overall weaker) profiles, 1,2,4-oxadiazoles (**3d**, **3ac**–**ad**) appear to stand out, with compound **3d** being the one that displayed activity against all five strains.

## 3. Materials and Methods

### 3.1. Compound Synthesis

NMR spectra were acquired with a 300 MHz Bruker Avance spectrometer (300.13 MHz for ^1^H and 75.5 MHz for ^13^C) in CDCl_3_ or DMSO-*d_6_* and were referenced to residual solvent proton signals (δ_H_ = 7.26 and 2.50, respectively) and solvent carbon signals (δ_C_ = 77.16 and 39.52, respectively). Multiplicities are abbreviated as follows: s = singlet, d = doublet, t = triplet, q = quartet, m = multiplet, br = broad, dd = doublet of doublets, dt = doublet of triplets, ddd = doublet/doublets of doublets; coupling constants, J, are reported in Hz. Mass spectra were acquired with an HRMS-ESI-qTOF spectrometer Nexera LCMS9030 or MaXis II Bruker Daltonic GmbH (electrospray ionization mode, positive ions detection). Flash column chromatography on silica (Merck, 230–400 mesh) was performed with a Biotage Isolera Prime instrument. TLC was performed on aluminum-backed pre-coated plates (0.25 mm) with silica gel 60 F_254_ with a suitable solvent system and was visualized using UV fluorescence.

#### 3.1.1. 4-[2-(Pyridin-2-yloxy)ethyl]-1-oxa-9-azaspiro[5.5]undecane Ditrifluoroacetate (**2a**)

To a 0 °C suspension of NaH (60% dispersion in mineral oil, 0.22 g, 5.6 mmol) in dry DMF (100 mL) a solution of *tert*-butyl 4-(2-hydroxyethyl)-1-oxa-9-azaspiro[5.5]udecan-9-carboxylate (**4**, 1.0 g, 3.3 mmol) in DMF (20 mL) was added under argon while stirring. After 30 min of stirring at 0 °C, a solution of 2-chloropyridin (5.0 mmol) in DMF (10 mL) was added. The reaction mixture was allowed to reach room temperature and was stirred at that temperature for 16 h. It was poured into water (200 mL) and the resulting mixture was extracted with ethyl acetate (3 × 200 mL). The combined organic extracts were washed with 5% aqueous citric acid, 5% aqueous NaHCO_3_ brine and filtered. The filtrate was dried over anhydrous Na_2_SO_4_, filtered and concentrated in vacuo. The residue was dissolved in DCM (10 mL), cooled to 0 °C and treated with trifluoroacetic acid (3 mL). After stirring at 0 °C over 6 h, the reaction mixture was evaporated to dryness, triturated with ether (3×), filtered and dried in vacuo. Yield: 0.56 g (1.11 mmol), 34%. ^1^H-NMR (300 MHz, DMSO-*d_6_*) δ 8.50 (br.s, 1H), 8.30 (br.s, 1H), 8.14 (ddd, *J* = 5.0, 2.0, 0.8 Hz, 1H), 7.69 (ddd, *J* = 8.4, 7.1, 2.0 Hz, 1H), 6.96 (ddd, *J* = 7.1, 5.1, 0.9 Hz, 1H), 6.79 (dt, *J* = 8.4, 0.9 Hz, 1H), 4.30 (t, *J* = 6.7 Hz, 2H), 3.71–3.64 (m, 1H), 3.50 (td, *J* = 12.3, 1.9 Hz, 1H), 3.12–3.00 (m, 3H), 2.92–2.78 (m, 1H), 2.37–2.29 (m, 1H), 1.93–1.79 (m, 1H), 1.72–1.54 (m, 6H), 1.42 (ddd, *J* = 14.9, 12.6, 4.2 Hz, 1H), 1.24–0.99 (m, 2H). ^13^C-NMR (75 MHz, CDCl_3_) δ 163.21, 158.35 (q, *J* = 37.4 Hz), 146.7, 139.3, 116.9, 115.4 (q, *J* = 290.2 Hz), 110.7, 68.1, 62.9, 60.1, 41.8, 35.6, 35.4, 32.1, 26.6, 25.6. MS *m/z* 278.4 (M + H^+^).

#### 3.1.2. 4-[(3-Ethyl-1,2,4-oxadiazol-5-yl)methyl]-1-oxa-9-azaspiro[5.5]undecane Hydrochloride (**2d**)

9-(*tert*-Butoxycarbonyl)-1-oxa-9-azaspiro[5.5]undec-4-yl]acetic acid (**5**, 8.0 g, 25.5 mmol) in THF (70 mL) was treated with *N*-methylmorpholine (3.09 g, 30.6 mmol) and the mixture was cooled to –30 °C. Isobutyl chloroformate (3.99 g, 29.3 mmol) was added dropwise and the mixture was left to stir at –30 °C for 30 min. *N*’-Hydroxypropanimidamide (2.18 g, 24.7 mmol) was added at –5 °C and the stirring continued at r. t. overnight. The reaction mixture was filtered and the filtrate was concentrated on a rotary evaporator. The residue was taken up in toluene (50 mL), TBAF (0.6 g) was added and the mixture was brought to reflux with the azeotropic removal of water and heated at reflux for 9 h. Upon cooling to r. t., toluene was removed in vacuo. The residue was dissolved in ethyl acetate and the solution was washed with 10% aqueous K_2_CO_3_ (2 × 20 mL). The organic phase was dried over Na_2_SO_4_, filtered and concentrated in vacuo. The residue was fractionated on a silica gel column eluted with 25–100% ethyl acetate in petroleum ether. Fractions containing the reaction product (according to TLC analysis) were pooled and concentrated in vacuo. The residue was treated with 4M HCl in 1,4-dioxane (15 mL) at 0 °C and the mixture was stirred for 6 h. The volatiles were removed in vacuo to afford the title compound. Yield: 6.34 g (21 mmol), 85%. m.p. 140–142 °C, ^1^H-NMR (300 MHz, DMSO-*d_6_*) δ 9.14 (br.s, 2H), 3.67 (dd, *J* = 11.7, 4.4 Hz, 1H), 3.56–3.46 (m, 1H), 3.04–2.97 (m, 3H), 2.84–2.78 (m, 3H), 2.68 (q, *J* = 7.5 Hz, 2H), 2.39–2.32 (m, 1H), 2.27–2.14 (m, 1H), 1.83–1.71 (m, 1H), 1.56 (d, *J* = 12.6 Hz, 4H), 1.21 (t, *J* = 7.5 Hz, 3H), 1.24–1.16 (m, 1H), 1.16–1.06 (m, 1H). ^13^C-NMR (75 MHz, DMSO-*d_6_*) δ 178.0, 171.1, 68.3, 59.8, 41.2, 40.3, 38.8, 35.0, 32.5, 31.5, 28.3, 25.3, 19.0, 11.1. MS *m/z* 266.4 (M + H^+^).

#### 3.1.3. *N*-Benzyl-*N*-cyclopropyl-1-oxa-9-azaspiro[5.5]undecan-4-amine Hydrochloride (**2ab**)

*Tert*-butyl 4-oxo-1-oxa-9-azaspiro[5.5]undecane-9-carboxylate (**6**, 2.0 g, 7.43 mmol) and cyclopropylamine (0.47 g, 8.15 mmol) in dichloromethane (50 mL) were treated with sodium triacetoxyborohydride (3.94 g, 18.6 mmol) with stirring. The reaction mixture was stirred overnight, poured into aqueous K_2_CO_3_ and extracted with dichloromethane (3 × 50 mL). The combined organic extracts were dried over anhydrous Na_2_SO_4_, filtered and concentrated in vacuo. The residue was taken up in dichloromethane (80 mL), treated with benzaldehyde (0.79 g, 7.43 mmol) and sodium triacetoxyborohydride (3.94 g, 18.6 mmol) with stirring. The reaction mixture was stirred overnight, poured into aqueous K_2_CO_3_ and extracted with dichloromethane (3 × 50 mL). The combined organic extracts were dried over anhydrous Na_2_SO_4_, filtered and concentrated in vacuo. The residue was fractionated on a silica gel column eluted with 0 → 10% MeOH in dichloromethane. Fractions containing the product (TLC and LCMS analysis) were pooled and concentrated in vacuo. The residue was treated with 4M HCl in 1,4-dioxane (6 mL) at 0 °C and the mixture was stirred for 6 h. The volatiles were removed on a rotary evaporator to give the title compound. Yield: 1.6 g (5.35 mmol), 72%; m.p. 221–223 °C; ^1^H-NMR (300 MHz, DMSO-*d_6_*) δ 11.22 (br.s, 1H), 9.15 (s, 2H), 7.72–7.62 (m, 2H), 7.48–7.39 (m, 3H), 4.40 (s, 2H), 3.83 (dd, *J* = 11.6, 4.0 Hz, 1H), 3.63–3.49 (m, 3H), 3.07–2.99 (m, 2H), 2.85–2.57 (m, 2H), 2.38–2.10 (m, 3H), 2.00–1.83 (m, 2H), 1.81–1.66 (m, 2H), 1.63–1.47 (m, 1H), 1.33–1.27 (m, 1H), 0.87–0.73 (m, 2H), 0.63–0.50 (m, 1H). ^13^C-NMR (75 MHz, DMSO-*d_6_*) δ 142.6, 132.0, 128.5, 126.5, 69.6, 62.8, 59.2, 59.2, 58.1, 57.7, 54.9, 54.4, 38.7, 35.8, 35.4, 35.0, 34.1, 33.7, 27.6, 27.2, 25.3, 5.4, 5.1, 4.5, 4.2. MS *m/z* 302.4 (M + H^+^).

#### 3.1.4. *N*-Cyclopropyl-*N*-1-oxa-9-azaspiro[5.5]undec-4-ylbenzamide Hydrochloride (**2af**)

*Tert*-butyl 4-oxo-1-oxa-9-azaspiro[5.5]undecane-9-carboxylate (**6**, 2.0 g, 7.43 mmol) and cyclopropylamine (0.47 g, 8.15 mmol) in dichloromethane (50 mL) was treated with sodium triacetoxyborohydride (3.94 g, 18.6 mmol) with stirring. The reaction mixture was stirred overnight, poured into aqueous K_2_CO_3_ and extracted with dichloromethane (3 × 50 mL). The combined organic extracts were dried over anhydrous Na_2_SO_4_, filtered and concentrated in vacuo. The residue was taken up in dichloromethane (80 mL), treated with Et_3_N (0.98 g, 9.66 mmol) and benzoyl chloride (1.15 g, 8.17 mmol). The reaction mixture was stirred at r. t. for 6 h, washed with water (3 × 20 mL), 5% aqueous HCl (3 × 20 mL) and 5% aqueous K_2_CO_3_ (3 × 20 mL). The organic phase was dried over anhydrous Na_2_SO_4_, filtered and concentrated in vacuo. The residue was treated with 4M HCl in 1,4-dioxane (6 mL) at 0 °C and the mixture was stirred for 6 h. The volatiles were removed on a rotary evaporator to give the title compound. Yield: 1.5 g (4.27 mmol), 58%. m.p. 194–196 °C, ^1^H-NMR (300 MHz, DMSO-*d_6_*) δ 9.05 (br.s, 1H), 7.48–7.44 (m, 2H), 7.43–7.38 (m, 3H), 4.41–4.31 (m, 1H), 3.79 (dd, *J* = 11.9, 3.7 Hz, 1H), 3.67–3.57 (m, 1H), 3.09–2.99 (m, 3H), 2.90–2.79 (m, 1H), 2.74–2.66 (m, 1H), 2.41–2.35 (m, 1H), 2.13–1.96 (m, 1H), 1.87–1.76 (m, 4H), 1.70–1.62 (m, 2H), 0.49 (d, *J* = 7.1 Hz, 2H), 0.40–0.28 (m, 3H). ^13^C-NMR (75 MHz, DMSO-*d_6_*) δ 171.8, 138.2, 129.2, 127.8, 127.1, 69.6, 60.1, 51.0, 38.9, 35.2, 30.3, 28.6, 25.3, 9.7, 9.5. MS *m/z* 316.4 (M + H^+^).

#### 3.1.5. 1-Cyclopropyl-3-(1-methylethyl)-1-(1-oxa-9-azaspiro[5.5]undec-4-yl)urea Dihydrochloride (**2ah**)

*Tert*-butyl 4-oxo-1-oxa-9-azaspiro[5.5]undecane-9-carboxylate (**6**, 2.0 g, 7.43 mmol) and cyclopropylamine (0.47 g, 8.15 mmol) in dichloromethane (50 mL) was treated with sodium triacetoxyborohydride (3.94 g, 18.6 mmol) with stirring. The reaction mixture was stirred overnight, poured into aqueous K_2_CO_3_ and extracted with dichloromethane (3 × 50 mL). The combined organic extracts were dried over anhydrous Na_2_SO_4_, filtered and concentrated in vacuo. The residue was taken up in 1,4-dioxane (80 mL) and treated with isopropyl isocyanate (0.7 g, 8.17 mmol). The reaction mixture was stirred at r. t. for 6 h, and washed with water (3 × 20 mL), 5% aqueous HCl (3 × 20 mL) and 5% aqueous K_2_CO_3_ (3 × 20 mL). The organic phase was dried over anhydrous Na_2_SO_4_, filtered and concentrated in vacuo. The residue was treated with 4M HCl in 1,4-dioxane (6 mL) at 0 °C and the mixture was stirred for 6 h. The volatiles were removed on a rotary evaporator to give the title compound. Yield: 2.13 g (5.79 mmol), 78%. m.p. 186–188 °C, ^1^H-NMR (300 MHz, DMSO-*d_6_*) δ 9.28–9.03 (m, 2H), 4.23 (br.s, 2H), 4.09–4.03 (m, 1H), 3.76–3.67 (m, 2H), 3.56–3.48 (m, 1H), 2.99 (br.s, 2H), 2.85–2.74 (m, 1H), 2.37–2.30 (m, 1H), 2.29–2.25 (m, 1H), 1.95–1.88 (m, 1H), 1.78–1.71 (m, 1H), 1.63–1.59 (m, 2H), 1.54–1.49 (m, 2H), 1.06 (d, *J* = 6.5 Hz, 6H), 0.83 (d, *J* = 5.0 Hz, 2H), 0.55 (d, *J* = 2.7 Hz, 2H). ^13^C-NMR (75 MHz, DMSO-*d_6_*) δ 158.3, 69.7, 62.3, 60.6, 50.4, 42.0, 40.5, 40.4, 39.1, 35.4, 31.1, 25.6, 25.4, 23.2, 9.2, 9.1. MS *m/z* 297.4 (M + H^+^).

#### 3.1.6. 1-Cyclopropyl-3-ethyl-1-(1-oxa-9-azaspiro[5.5]undec-4-yl)urea Dihydrochloride (**2ai**)

The compound was prepared analogously to compound **2ah** using ethyl isocyanate. Yield: 2.32 g (6.54 mmol), 88%. m.p. 197–199 °C ^1^H-NMR (300 MHz, DMSO-*d_6_*) δ 9.22–9.00 (m, 2H), 4.13–4.03 (m, 1H), 3.73–3.67 (m, 1H), 3.57–3.48 (m, 1H), 3.10–2.93 (m, 5H), 2.86–2.73 (m, 1H), 2.37–2.21 (m, 2H), 2.00–1.85 (m, 1H), 1.81–1.69 (m, 2H), 1.63–1.47 (m, 4H), 1.00 (t, *J* = 7.1 Hz, 3H), 0.85–0.78 (m, 2H), 0.55 (d, *J* = 2.3 Hz, 2H). ^13^C-NMR (75 MHz, DMSO-*d_6_*) δ 159.1, 69.8, 60.6, 50.5, 40.5, 39.2, 35.5, 35.1, 31.2, 25.6, 16.0, 9.2. MS *m/z* 283.4 (M + H^+^).

#### 3.1.7. 1-Cyclopropyl-3-ethyl-1-(1-oxa-9-azaspiro[5.5]undec-4-yl)urea Dihydrochloride (**2aj**)

The compound was prepared analogously to compound **2af**. Yield: 0.85 g (2.94 mmol), 68%. m.p. 178–180 °C, ^1^H-NMR (300 MHz, DMSO-*d_6_*) δ 9.20–9.04 (m, 2H), 4.26–4.15 (m, 1H), 3.72 (dd, *J* = 11.9, 4.7 Hz, 1H), 3.59–3.50 (m, 1H), 3.07–2.92 (m, 3H), 2.86–2.73 (m, 1H), 2.61–2.54 (m, 1H), 2.36 (dd, *J* = 14.5, 2.0 Hz, 1H), 2.08 (s, 3H), 2.03–1.92 (m, 1H), 1.83–1.72 (m, 2H), 1.63–1.51 (m, 4H), 0.85–0.79 (m, 2H), 0.76–0.71 (m, 2H). ^13^C-NMR (75 MHz, DMSO-*d_6_*) δ 173.2, 69.8, 60.4, 50.4, 39.8, 39.1, 39.0, 35.4, 30.5, 28.7, 25.4, 23.8, 9.2. MS *m/z* 254.4 (M + H^+^).

#### 3.1.8. 7-Chloro-1-cyclopropyl-6-fluoro-4-oxo-1,4-dihydroquinoline-3-carboxylic Acid Ethyl Ester (**8**)

7-Chloro-1-cyclopropyl-6-fluoro-4-oxo-1,4-dihydroquinoline-3-carboxylic acid (**7**, 17.8 mmol) was dissolved in DMF (300 mL) and treated with K_2_CO_3_ (28.4 mmol). After 30 min of stirring, ethyl bromide (135 mmol) was added and stirring continued for 10 h. DMF was removed in vacuum and the residue was washed with water (2 × 50 mL) and filtered. The residue was air-dried and then dried in vacuo [21]. Yield—5 g (91%), white solid, m.p. 121–123°. ^1^H-NMR (300 MHz, DMSO-*d_6_*) δ 8.47 (s, 1H), 8.28 (d, *J* = 4.6 Hz, 1H), 7.98 (d, *J* = 8.6 Hz, 1H), 4.22 (d, *J* = 6.5 Hz, 2H), 3.67 (s, 1H), 1.28 (s, 5H), 1.12 (s, 2H). MS *m/z* 311.6 (M + H^+^).

#### 3.1.9. 8-Chloro-6-cyclopropyl-2,2-diacetoxy-9-fluoro-4-oxo-4,6-dihydro-2H-[1,3,2]-dioxaborinino[5,4-c]-quinolin-1-ium-2-uide (**9**)

The reaction mixture consisting of H_3_BO_3_ (48 mmol), Ac_2_O (146 mmol) and ZnCl_2_ (0.4 mmol) was stirred at r. t. for 30 min. 7-Chloro-1-cyclopropyl-6-fluoro-4-oxo-1,4-dihydroquinoline-3-carboxylic acid ethyl ester (**8**, 5 g) was added and the stirring continued at 60 °C for 2 h. The volatiles were removed in vacuo. The residue was washed with ethyl acetate (50 mL) and water (50 mL) and dried in vacuo. Yield—6.27 g (94.7%), white solid, m.p. 113–115 °C. ^1^H-NMR (300 MHz, DMSO-*d_6_*) δ 9.24–9.22 (m, 1H), 8.87 (d, *J* = 6.1 Hz, 1H), 8.38 (d, *J* = 8.6 Hz, 1H), 4.24–4.14 (m, 1H), 1.91 (s, 6H), 1.44 (d, *J* = 6.5 Hz, 4H). ^13^C-NMR (75 MHz, DMSO-*d_6_*) δ 171.2, 169.1 (d, *J* = 3.7 Hz), 159.2, 156.2 (d, *J* = 253.4 Hz), 149.4, 138.8 (d, *J* = 1.4 Hz), 130.0 (d, *J* = 20.5 Hz), 122.4, 121.1 (d, *J* = 8.5 Hz), 110.9 (d, *J* = 23.8 Hz), 108.4, 38.6, 22.8, 7.9. MS *m/z* 411.6 (M + H^+^).

#### 3.1.10. General Procedure for the Preparation of Compounds **3a**–**aj**

Compound **9** (0.24 mmol) was dissolved in acetonitrile (10 mL) and treated, with stirring, with spirocyclic amine **2** (0.47 mmol) and triethylamine (0.28 mmol). The stirring continued at 60 °C for 10 h. The volatiles were removed in vacuo. The residue was fractionated on a silica gel column eluted with 0 → 20% methanol in dichloromethane. Fractions containing the product (by TLC analysis) were pooled and concentrated in vacuo. The residues was dissolved in 2% aqueous NaOH and left to stir at r. t. overnight. The reaction mixture was acidified with 5% aqueous citric acid to pH 4–5. The resulting precipitate was filtered off, washed with water and air-dried.

##### 1-Cyclopropyl-6-fluoro-4-oxo-7-(4-(2-(pyridine-2-yloxy)-ethyl)-1-oxa-9-azaspiro[5.5]undec-9-yl)-1,4-dihydroquinoline-3-carboxylic Acid (**3a**)

Yield—100 mg (44%), white solid, m.p. 170–172 °C. ^1^H-NMR (300 MHz, CDCl_3_) δ 15.10 (s, 1H), 8.74 (s, 1H), 8.14 (d, *J* = 3.6 Hz, 1H), 7.97 (d, *J* = 13.1 Hz, 1H), 7.61–7.53 (m, 1H), 7.37 (d, *J* = 7.1 Hz, 1H), 6.93–6.82 (m, 1H), 6.72 (d, *J* = 8.3 Hz, 1H), 4.35 (t, *J* = 6.4 Hz, 2H), 3.80 (dd, *J* = 11.5, 4.6 Hz, 1H), 3.64 (t, *J* = 11.4 Hz, 1H), 3.55–3.39 (m, 3H), 3.32 (td, *J* = 11.7, 2.7 Hz, 1H), 3.11 (t, *J* = 11.3 Hz, 1H), 2.40 (br.d, *J* = 13.1 Hz, 1H), 2.04–1.93 (m, 1H), 1.92–1.76 (m, 2H), 1.74–1.69 (m, 3H), 1.67–1.52 (m, 2H), 1.42–1.34 (m, 2H), 1.33–1.27 (m, 1H), 1.26–1.16 (m, 3H); ^13^C-NMR (75 MHz, CDCl_3_) δ 177.0 (d, *J* = 2.5 Hz), 167.2, 163.9, 153.7 (d, *J* = 251.3 Hz), 147.2, 146.9, 146.5 (d, *J* = 10.3 Hz), 139.2, 138.7, 119.2 (d, *J* = 7.9 Hz), 116.8, 112.0 (d, *J* = 23.4 Hz), 111.1, 107.8, 104.9 (d, *J* = 3,3 Hz), 69.8, 63.1, 60.8, 45.7, 45.7, 45.6, 43.1, 39.2, 36.3, 35.4, 32.9, 29.3, 27.4, 8.3, 8.2; HRMS (ESI) *m/z* calculated for C_29_H_32_FN_3_O_5_ [M + H^+^] 522.2399, found 522.2422.

##### 1-Cyclopropyl-6-fluoro-4-oxo-7-(4-(4-methylbenzyl)-1-oxa-9-azaspiro[5.5]undec-9-yl)-1,4-dihydroquinoline-3-carboxylic Acid (**3b**)

Yield—47 mg (22%), yellow solid, m.p. 215–217 °C. ^1^H-NMR (300 MHz, CDCl_3_) δ 15.05 (s, 1H), 8.71 (s, 1H), 7.93 (d, *J* = 13.1 Hz, 1H), 7.37 (d, *J* = 6.7 Hz, 1H), 7.06 (dd, *J* = 19.7, 7.4 Hz, 4H), 3.82–3.71 (m, 1H), 3.63–3.28 (m, 5H), 3.11 (t, *J* = 11.6 Hz, 1H), 2.48 (d, *J* = 6.6 Hz, 2H), 2.40–2.25 (m, 4H), 1.95–1.69 (m, 3H), 1.65–1.50 (m, 3H), 1.42–1.32 (m, 2H), 1.30–1.14 (m, 4H); ^13^C-NMR (75 MHz, CDCl_3_) δ 177.1 (d, *J* = 2.5 Hz), 167.3, 153.8 (d, *J* = 251.4 Hz), 147.4, 146.5 (d, *J* = 10.3 Hz), 139.2, 136.7, 135.6, 129.1, 119.4 (d, *J* = 7.9 Hz), 112.2 (d, *J* = 23.5 Hz), 108.0, 105.0 (d, *J* = 2.3 Hz), 69.9, 60.9, 45.9, 45.8, 45.8, 45.7, 43.4, 43.1, 39.2, 35.4, 32.7, 32.5, 29.3, 21.1, 8.4, 8.3; HRMS (ESI) *m/z* calculated for C_30_H_33_FN_2_O_4_ [M + H^+^] 505.2497, found 505.2517.

##### 1-Cyclopropyl-6-fluoro-4-oxo-7-(4-(2-(6-methylpyridin-2-yloxy)-ethyl)-1-oxa-9-azaspiro[5.5]undec-9-yl)-1,4-dihydroquinoline-3-carboxylic Acid (**3c**)

Yield—60 mg (27%), white solid, m.p. 150–152 °C. ^1^H-NMR (300 MHz, CDCl_3_) δ 15.14 (s, 1H), 8.74 (s, 1H), 7.97 (d, *J* = 13.1 Hz, 1H), 7.46 (t, *J* = 7.7 Hz, 1H), 7.37 (d, *J* = 7.2 Hz, 1H), 6.71 (d, *J* = 7.2 Hz, 1H), 6.51 (d, *J* = 8.2 Hz, 1H), 4.31 (t, *J* = 6.3 Hz, 2H), 3.80 (dd, *J* = 11.7, 4.3 Hz, 1H), 3.64 (t, *J* = 11.5 Hz, 1H), 3.56–3.38 (m, 3H), 3.37–3.26 (m, 1H), 3.10 (t, *J* = 11.2 Hz, 1H), 2.43 (s, 3H), 2.41 (br.d, *J* = 15.7 Hz, 1H), 2.07–1.94 (m, 1H), 1.92–1.81 (m, 1H), 1.77–1.65 (m, 5H), 1.62–1.52 (m, 1H), 1.42–1.34 (m, 2H), 1.33–1.25 (m, 1H), 1.24–1.15 (m, 3H); ^13^C-NMR (75 MHz, CDCl_3_) δ 177.1 (d, *J* = 1.9 Hz), 167.3, 163.4, 156,4, 153.8 (d, *J* = 251.3 Hz), 147.3, 146.6 (d, *J* = 10.4 Hz), 139.2, 138.9, 119.3 (d, *J* = 7.1 Hz), 115.9, 112.1 (d, *J* = 23.6 Hz), 107.0, 104.9 (d, *J* = 3.5 Hz), 69.8, 62.9, 60.9, 45.8, 45.7, 45.7, 43.2, 39.3, 36.5, 35.4, 32.7, 29.3, 27.3, 24.3, 8.3, 8.3; HRMS (ESI) *m/z* calculated for C_30_H_34_FN_3_O_5_ [M + H^+^] 536.2555, found 536.2578.

##### 1-Cyclopropyl-7-(4-(3-ethyl(1,2,4)oxadiazol-5-ylmethyl)-1-oxa-9-azaspiro[5.5]undec-9-yl)-6-fluoro-4-oxo-1,4-dihydroquinoline-3-carboxylic Acid (**3d**)

Yield—125 mg (59%), white solid, m.p. 141–143 °C. ^1^H-NMR (300 MHz, CDCl_3_) δ 15.03 (s, 1H), 8.71 (s, 1H), 7.94 (d, *J* = 13.1 Hz, 1H), 7.42 (d, *J* = 6.3 Hz, 1H), 3.81 (dd, *J* = 11.8, 4.4 Hz, 1H), 3.64 (t, *J* = 11.6 Hz, 1H), 3.55–3.25 (m, 4H), 3.12 (t, *J* = 11.3 Hz, 1H), 2.84–2.67 (m, 4H), 2.44–2.24 (m, 2H), 1.94–1.81 (m, 1H), 1.78–1.56 (m, 4H), 1.43–1.24 (m, 7H), 1.23–1.13 (m, 2H); ^13^C-NMR (75 MHz, CDCl_3_) δ 177.7, 176.8 (d, *J* = 2.5 Hz), 171.6, 167.0, 153.6 (d, *J* = 251.4 Hz), 147.2, 146.2 (d, *J* = 10.3 Hz), 139.1, 119.1 (d, *J* = 7.9 Hz), 111.8 (d, *J* = 23.6 Hz), 107.7, 104.9 (d, *J* = 2.8 Hz), 69.7, 60.3, 45.5, 45.5, 42.4, 39.0, 35.4, 33.7, 32.2, 29.3, 29.1, 19.7, 11.3, 8.2; HRMS (ESI) *m/z* calculated for C_27_H_31_FN_4_O_5_ [M + H^+^] 511.2351, found 511.2369.

##### 1-Cyclopropyl-6-fluoro-7-(1-oxa-9-azaspiro[5.5]undec-9-yl)-4-oxo-1,4-dihydroquinoline-3-carboxylic Acid (**3e**)

Yield—83 mg (50%), yellow solid, m.p. 190–192 °C. ^1^H-NMR (300 MHz, CDCl_3_) δ 15.06 (s, 1H), 8.70 (s, 1H), 7.92 (d, *J* = 13.2 Hz, 1H), 7.36 (d, *J* = 7.2 Hz, 1H), 3.69 (t, *J* = 5.1 Hz, 2H), 3.56–3.39 (m, 3H), 3.22 (t, *J* = 11.3 Hz, 2H), 2.15–2.03 (m, 2H), 1.76–1.63 (m, 4H), 1.61–1.49 (m, 4H), 1.42–1.31 (m, 2H), 1.23–1.14 (m, 2H); ^13^C-NMR (75 MHz, CDCl_3_) δ 177.2 (d, *J* = 2.7 Hz), 167.2, 153.9 (d, *J* = 251.3 Hz), 147.3, 146.6 (d, *J* = 10.4 Hz), 139.3, 119.4 (d, *J* = 7.9 Hz), 112.2 (d, *J* = 23.6 Hz), 108.1, 104.9 (d, *J* = 3.7 Hz), 69.4, 61.1, 45.8, 45.7, 36.2, 35.4, 34.1, 26.2, 18.9, 8.3; HRMS (ESI) *m/z* calculated for C_22_H_25_FN_2_O_4_ [M + H^+^] 401.1871, found 401.1889.

##### 1-Cyclopropyl-6-fluoro-4-oxo-7-(4-(pyridin-4-yloxy)-1-oxa-9-azaspiro[5.5]undec-9-yl)-1,4-dihydroquinoline-3-carboxylic Acid (**3f**)

Yield—120 mg (59%), beige solid, m.p. 138–140 °C. ^1^H-NMR (300 MHz, CDCl_3_) δ 15.09 (s, 1H), 8.71 (s, 1H), 8.42 (d, *J* = 4.6 Hz, 2H), 7.93 (d, *J* = 13.0 Hz, 1H), 7.36 (d, *J* = 7.1 Hz, 1H), 6.81 (d, *J* = 5.4 Hz, 2H), 4.83–4.69 (m, 1H), 4.04–3.94 (m, 1H), 3.80–3.69 (m, 1H), 3.58–3.40 (m, 3H), 3.33–3.13 (m, 2H), 2.53 (br.s, 1H), 2.20–2.09 (m, 3H), 2.02–1.94 (m, 1H), 1.87–1.75 (m, 3H), 1.43–1.32 (m, 2H), 1.22–1.14 (m, 2H); ^13^C-NMR (75 MHz, CDCl_3_) δ 177.1 (d, *J* = 2.6 Hz), 167.0, 163.8, 153.7 (d, *J* = 251.3 Hz), 150.5, 147.3, 146.3 (d, *J* = 10.4 Hz), 139.1, 119.5 (d, *J* = 7.9 Hz), 112.2 (d, *J* = 23.6 Hz), 111.1, 108.1, 104.8 (d, *J* = 3.5 Hz), 70.4, 70.2, 58.1, 45.6, 45.6, 45.5, 45.4, 40.5, 36.2, 35.3, 33.1, 31.1, 8.2; HRMS (ESI) *m/z* calculated for C_27_H_28_FN_3_O_5_ [M + H^+^] 494.2086, found 494.2107.

##### 1-Cyclopropyl-6-fluoro-7-(4-ethoxy-1-oxa-9-azaspiro[5.5]undec-9-yl)-4-oxo-1,4-dihydroquinoline-3-carboxylic Acid (**3g**)

Yield—160 mg (86%), white solid, m.p. 172–174 °C. ^1^H-NMR (300 MHz, CDCl_3_) δ 15.06 (s, 1H), 8.71 (s, 1H), 7.93 (d, *J* = 13.1 Hz, 1H), 7.38 (d, *J* = 6.9 Hz, 1H), 3.96–3.83 (m, 1H), 3.73–3.60 (m, 2H), 3.58–3.39 (m, 5H), 3.28 (t, *J* = 10.7 Hz, 1H), 3.15 (t, *J* = 11.5 Hz, 1H), 2.18–2.07 (m, 1H), 2.05–1.91 (m, 2H), 1.91–1.79 (m, 2H), 1.78–1.66 (m, 1H), 1.61–1.44 (m, 2H), 1.42–1.33 (m, 2H), 1.26–1.16 (m, 5H); ^13^C-NMR (75 MHz, CDCl_3_) δ 177.2 (d, *J* = 2.5 Hz), 167.2, 153.9 (d, *J* = 251.4 Hz), 147.4, 146.3 (d, *J* = 10.1 Hz), 139.2, 119.6 (d, *J* = 7.9 Hz), 112.3 (d, *J* = 23.6 Hz), 108.1, 105.2 (d, *J* = 2.4 Hz), 71.3, 70.7, 63.3, 59.1, 45.9, 45.9, 45.8, 45.8, 42.0, 37.4, 35.4, 32.4, 32.1, 15.7, 8.4; HRMS (ESI) *m/z* calculated for C_24_H_29_FN_2_O_5_ [M + H^+^] 445.2133, found 445.2148.

##### 1-Cyclopropyl-6-fluoro-4-oxo-7-(4-(2-(pyrimidin-2-yloxy)-ethyl)-1-oxa-9-azaspiro[5.5]undec-9-yl)-1,4-dihydroquinoline-3-carboxylic Acid (**3i**)

Yield—135 mg (62%), white solid, m.p. 182–184 °C. ^1^H-NMR (300 MHz, CDCl_3_) δ 15.11 (s, 1H), 8.73 (s, 1H), 8.51 (d, *J* = 4.7 Hz, 2H), 7.95 (d, *J* = 13.1 Hz, 1H), 7.36 (d, *J* = 7.1 Hz, 1H), 6.94 (t, *J* = 4.7 Hz, 1H), 4.42 (t, *J* = 6.2 Hz, 2H), 3.80 (dd, *J* = 11.6, 4.5 Hz, 1H), 3.63 (t, *J* = 11.5 Hz, 1H), 3.55–3.39 (m, 3H), 3.38–3.26 (m, 1H), 3.11 (t, *J* = 11.1 Hz, 1H), 2.39 (br.d, *J* = 13.7 Hz, 1H), 2.04 (dd, *J* = 11.9, 6.7 Hz, 1H), 1.83 (dd, *J* = 11.7, 4.0 Hz, 1H), 1.79–1.72 (m, 3H), 1.71–1.62 (m, 2H), 1.61–1.53 (m, 1H), 1.41–1.34 (m, 2H), 1.33–1.25 (m, 1H), 1.26–1.21 (m, 1H), 1.22–1.15 (m, 2H); ^13^C-NMR (75 MHz, CDCl_3_) δ 176.4 (d, *J* = 2.6 Hz), 166.5, 164.5, 158.6, 153.0 (d, *J* = 251.3 Hz), 146.5, 145.7 (d, *J* = 10.4 Hz), 138.5, 118.6 (d, *J* = 7.9 Hz), 114.3, 111.5 (d, *J* = 23.6 Hz), 107.3, 104.1 (d, *J* = 3.5 Hz), 69.0, 64.0, 60.0, 45.0, 45.0, 44.9, 42.3, 38.5, 35.4, 34.6, 31.8, 28.6, 26.4, 7.5; HRMS (ESI) *m/z* calculated for C_28_H_31_FN_4_O_5_ [M + Na^+^] 545.2171, found 545.2190.

##### 1-Cyclopropyl-7-(4-(2-(3,6-dimethylpyrazin-2-yloxy)-ethyl)-1-oxa-9-azaspiro[5.5]undec-9-yl) -6-fluoro-4-oxo-1,4-dihydroquinoline-3-carboxylic Acid (**3h**)

Yield—50 mg (53%), pale brown solid, m.p. 87–89 °C. ^1^H-NMR (300 MHz, CDCl_3_) δ 15.10 (s, 1H), 8.71 (s, 1H), 7.93 (d, *J* = 13.1 Hz, 1H), 7.83 (s, 1H), 7.36 (d, *J* = 7.2 Hz, 1H), 4.37 (t, *J* = 6.4 Hz, 2H), 3.81 (dd, *J* = 11.8, 4.6 Hz, 1H), 3.69–3.60 (m, 1H), 3.55–3.47 (m, 2H), 3.45–3.38 (m, 1H), 3.31 (td, *J* = 11.6, 2.8 Hz, 1H), 3.16–3.06 (m, 1H), 2.40 (s, 3H), 2.45–2.36 (m, 1H), 2.38 (s, 3H), 2.00–1.94 (m, 1H), 1.84 (dd, *J* = 11.7, 4.7 Hz, 1H), 1.78–1.73 (m, 2H), 1.73–1.69 (m, 2H), 1.65 (d, *J* = 8.9 Hz, 1H), 1.61–1.53 (m, 1H), 1.39–1.35 (m, 2H), 1.32–1.22 (m, 2H), 1.20–1.17 (m, 2H); ^13^C-NMR (75 MHz, CDCl_3_) δ 177.1 (d, *J* = 2.7 Hz), 167.3, 157.8, 153.8 (d, *J* = 251.3 Hz), 147.8, 147.4, 146.6 (d, *J* = 10.4 Hz), 140.8, 139.2, 134.1, 119.4 (d, *J* = 7.9 Hz), 112.2 (d, *J* = 23.6 Hz), 108.0, 104.9 (d, *J* = 3.4 Hz), 69.8, 63.4, 60.9, 45.8, 45.8, 45.7, 45.7, 43.2, 39.3, 36.2, 35.4, 32.8, 29.3, 27.6, 20.8, 18.8, 8.4, 8.3; HRMS (ESI) *m/z* calculated for C_30_H_35_FN_4_O_5_ [M + H^+^] 551.2664, found 551.2673.

##### 1-Cyclopropyl-6-fluoro-4-oxo-7-(4-(2-(5-trifluoromethylpyridin-2-yloxy)-ethyl)-1-oxa-9-azaspiro[5.5]undec-9-yl)-1,4-dihydroquinoline-3-carboxylic Acid (**3j**)

Yield—65 mg (29%), white solid, m.p. 95–97 °C. ^1^H-NMR (300 MHz, CDCl_3_) δ 15.09 (s, 1H), 8.74 (s, 1H), 8.42 (s, 1H), 7.97 (d, *J* = 13.2 Hz, 1H), 7.77 (d, *J* = 8.0 Hz, 1H), 7.37 (d, *J* = 7.1 Hz, 1H), 6.81 (d, *J* = 8.7 Hz, 1H), 4.42 (t, *J* = 6.2 Hz, 2H), 3.87–3.76 (m, 1H), 3.64 (t, *J* = 11.7 Hz, 1H), 3.56–3.40 (m, 3H), 3.32 (t, *J* = 10.9 Hz, 1H), 3.11 (t, *J* = 11.6 Hz, 1H), 2.40 (br.d, *J* = 14.2 Hz, 1H), 2.00–1.91 (m, 1H), 1.89–1.81 (m, 1H), 1.77–1.69 (m, 4H), 1.66–1.62 (m, 1H), 1.61–1.53 (m, 1H), 1.42–1.30 (m, 3H), 1.26–1.15 (m, 3H); ^13^C-NMR (75 MHz, CDCl_3_) δ 177.2 (d, *J* = 2.6 Hz), 167.3, 165.9 (q, *J* = 0.8 Hz), 153.9 (d, *J* = 251.3 Hz), 147.4, 146.6 (d, *J* = 10.4 Hz), 145.1 (q, *J* = 4.4 Hz), 139.3, 135.8 (q, *J* = 6.0, 2.9 Hz), 124.1 (q, *J* = 542.2, 271.1 Hz), 120.1 (q, *J* = 65.1, 32.0 Hz), 119.5 (d, *J* = 7.9 Hz), 112.3 (d, *J* = 23.6 Hz), 111.4, 108.1, 105.0 (d, *J* = 3.5 Hz), 69.8, 64.1, 60.8, 45.9, 45.8, 45.7, 45.7, 43.2, 39.3, 36.2, 35.4, 32.8, 29.4, 27.4, 8.4, 8.3; HRMS (ESI) *m/z* calculated for C_30_H_31_FN_4_O_5_ [M + H^+^] 547.2351, found 547.2039.

##### 1-Cyclopropyl-7-(4-(2-(2-cyclopropyl-6,7-dihydro-5H-cyclopentapyrimidin-4-yloxy)-ethyl)-1-oxa-9-azaspiro[5.5]undec-9-yl)-6-fluoro-4-oxo-1,4-dihydroquinoline-3-carboxylic Acid (**3k**)

Yield—97 mg (39%), white solid, m.p. 97–99 °C. ^1^H-NMR (300 MHz, CDCl_3_) δ 15.13 (s, 1H), 8.74 (s, 1H), 7.97 (d, *J* = 13.1 Hz, 1H), 7.37 (d, *J* = 7.1 Hz, 1H), 4.39 (t, *J* = 6.2 Hz, 2H), 3.81 (dd, *J* = 11.4, 3.9 Hz, 1H), 3.64 (t, *J* = 11.7 Hz, 1H), 3.57–3.38 (m, 3H), 3.31 (t, *J* = 10.6 Hz, 1H), 3.09 (t, *J* = 11.5 Hz, 1H), 2.90 (t, *J* = 7.6 Hz, 2H), 2.76 (t, *J* = 7.3 Hz, 2H), 2.40 (br.d, *J* = 13.3 Hz, 1H), 2.16–2.04 (m, 3H), 1.93–1.82 (m, 2H), 1.76–1.54 (m, 6H), 1.37 (br.d, *J* = 5.8 Hz, 2H), 1.33–1.23 (m, 2H), 1.23–1.17 (m, 2H), 1.11–1.03 (m, 2H), 0.98 (br.d, *J* = 7.6 Hz, 2H); ^13^C-NMR (75 MHz, CDCl_3_) δ 176.9 (d, *J* = 2.3 Hz), 174.4, 170.6, 167.1, 165.6, 153.7 (d, *J* = 251.4 Hz), 147.2, 146.4 (d, *J* = 10.4 Hz), 139.2, 119.1 (d, *J* = 7.6 Hz), 116.3, 111.9 (d, *J* = 23.8 Hz), 107.8, 104.8 (d, *J* = 2.9 Hz), 69.7, 63.0, 60.8, 45.7, 45.6, 45.6, 43.1, 39.2, 36.1, 35.4, 34.1, 32.8, 29.3, 27.5, 26.4, 21.9, 17.8, 10.0, 8.2; HRMS (ESI) *m/z* calculated for C_34_H_39_FN_4_O_5_ [M + H^+^] 603.2977, found 603.3007.

##### 1-Cyclopropyl-6-fluoro-7-(4-(2-(3-methylpyrazin-2-yloxy)-ethyl)-1-oxa-9-azaspiro[5.5]undec-9-yl)-4-oxo-1,4-dihydroquinoline-3-carboxylic Acid (**3l**)

Yield—55 mg (25%), white solid, m.p. 86–88 °C. ^1^H-NMR (300 MHz, CDCl_3_) δ 15.08 (s, 1H), 8.72 (s, 1H), 8.01–7.87 (m, 3H), 7.36 (d, *J* = 7.2 Hz, 1H), 4.38 (t, *J* = 6.6 Hz, 2H), 3.81 (dd, *J* = 11.8, 4.4 Hz, 1H), 3.65 (t, *J* = 11.3 Hz, 1H), 3.56–3.47 (m, 2H), 3.41 (br.s, 1H), 3.32 (td, *J* = 11.7, 2.9 Hz, 1H), 3.18–3.05 (m, 1H), 2.46 (s, 3H), 2.50–2.35 (m, 1H), 1.98–1.81 (m, 2H), 1.77–1.66 (m, 5H), 1.63–1.55 (m, 1H), 1.41–1.35 (m, 2H), 1.35–1.29 (m, 1H), 1.28–1.23 (m, 1H), 1.21–1.16 (m, 2H); ^13^C-NMR (75 MHz, CDCl_3_) δ 177.1 (d, *J* = 2.6 Hz), 167.2, 158.5, 153.7 (d, *J* = 251.3 Hz), 147.3, 146.5 (d, *J* = 10.4 Hz), 144.8, 139.1, 138.1, 135.5, 119.4 (d, *J* = 7.9 Hz), 112.2 (d, *J* = 23.6 Hz), 108.0, 104.8 (d, *J* = 3.5 Hz), 69.7, 63.6, 60.7, 45.7, 45.7, 45.6, 45.6, 43.0, 39.2, 36.0, 35.3, 32.7, 29.2, 27.6, 19.4, 8.3, 8.2; HRMS (ESI) *m/z* calculated for C_29_H_33_FN_4_O_5_ [M + H^+^] 537.2508, found 537.2531.

##### 7-(4-tert-Butoxycarbonylamino-1-oxa-9-azaspiro[5.5]undec-9-yl)-1-cyclopropyl-6-fluoro-4-oxo-1,4-dihydroquinoline-3-carboxylic Acid (**3m**)

Yield—110 mg (51%), white solid, m.p. 246–248 °C. ^1^H-NMR (300 MHz, CDCl_3_) δ 15.01 (s, 1H), 8.73 (s, 1H), 7.97 (d, *J* = 13.1 Hz, 1H), 7.42 (d, *J* = 6.9 Hz, 1H), 4.38 (br.s, 1H), 3.93–3.77 (m, 2H), 3.68 (t, *J* = 11.6 Hz, 1H), 3.56–3.41 (m, 3H), 3.40–3.28 (m, 1H), 3.15 (t, *J* = 11.2 Hz, 1H), 2.34 (br.d, *J* = 13.8 Hz, 1H), 2.01–1.86 (m, 3H), 1.80–1.69 (m, 2H), 1.44 (s, 9H), 1.43–1.35 (m, 3H), 1.26–1.16 (m, 3H); ^13^C-NMR (75 MHz, CDCl_3_) δ 177.1 (d, *J* = 2.6 Hz), 167.2, 155.2, 153.8 (d, *J* = 251.4 Hz), 147.4, 146.3 (d, *J* = 10.2 Hz), 139.2, 119.5 (d, *J* = 7.8 Hz), 112.2 (d, *J* = 23.6 Hz), 108.0, 105.1 (d, *J* = 2.5 Hz), 79.7, 60.1, 45.8, 45.7, 45.6, 45.6, 43.6, 43.1, 39.0, 35.4, 33.5, 29.5, 28.5, 8.3; HRMS (ESI) *m/z* calculated for C_27_H_34_FN_3_O_6_ [M + H^+^] 516.2504, found 516.2486.

##### 7-(4-Amino-1-oxa-9-azaspiro[5.5]undec-9-yl)-1-cyclopropyl-6-fluoro-4-oxo-1,4-dihydroquinoline-3-carboxylic Acid (**3n**)

Yield—60 mg (35%), brown solid, m.p. 131–133 °C. ^1^H-NMR (300 MHz, D_2_O) δ 8.53 (s, 1H), 7.32 (br.s, 1H), 7.15 (d, *J* = 6.3 Hz, 1H), 4.00 (br.s, 1H), 3.97–3.87 (m, 1H), 3.82 (br.s, 1H), 3.70–3.36 (m, 3H), 3.33–3.09 (m, 2H), 2.51–2.35 (m, 1H), 2.25–2.11 (m, 2H), 2.00 (br.s, 1H), 1.92–1.73 (m, 3H), 1.70–1.60 (m, 1H), 1.46 (br.s, 2H), 1.15 (br.s, 2H); ^13^C-NMR (75 MHz, D_2_O) δ 175.8, 169.4, 153.6 (d, *J* = 251.4 Hz), 148.1, 146.2 (d, *J* = 7.5 Hz), 139.3, 117.6 (d, *J* = 3.5 Hz), 110.6 (d, *J* = 24.3 Hz), 106.1, 105.9, 72.1, 59.6, 45.8, 45.8, 45.7, 45.7, 44.8, 39.6, 38.3, 36.6, 30.5, 29.1, 8.0; HRMS (ESI) *m/z* calculated for C_22_H_26_FN_3_O_4_ [M + H^+^] 416.1980, found 416.1996.

##### 7-(4-(Benzyloxycarbonylaminomethyl)-1-oxa-9-azaspiro[5.5]undec-9-yl)-1-cyclopropyl-6-fluoro-4-oxo-1,4-dihydroquinoline-3-carboxylic Acid (**3o**)

Yield—220 mg (93%), white solid, m.p. 102–104 °C. ^1^H-NMR (300 MHz, CDCl_3_) δ 15.09 (s, 1H), 8.73 (s, 1H), 7.96 (d, *J* = 13.1 Hz, 1H), 7.42–7.31 (m, 6H), 5.10 (s, 2H), 4.92 (br.s, 1H), 3.81 (dd, *J* = 11.6, 4.3 Hz, 1H), 3.61 (t, *J* = 11.9 Hz, 1H), 3.56–3.39 (m, 3H), 3.37–3.26 (m, 1H), 3.17–3.02 (m, 3H), 2.34 (br.d, *J* = 13.2 Hz, 1H), 1.93 (d, *J* = 13.8 Hz, 1H), 1.84 (dd, *J* = 11.5, 3.9 Hz, 1H), 1.71 (d, *J* = 14.6 Hz, 2H), 1.61 (d, *J* = 13.8 Hz, 2H), 1.42–1.33 (m, 2H), 1.28–1.10 (m, 4H); ^13^C-NMR (75 MHz, CDCl_3_) δ 176.9 (d, *J* = 2.6 Hz), 167.1, 156.6, 153.6 (d, *J* = 251.3 Hz), 147.2, 146.3 (d, *J* = 10.3 Hz), 139.1, 136.5, 128.5, 128.1, 128.0, 119.1 (d, *J* = 7.9 Hz), 111.9 (d, *J* = 23.6 Hz), 107.8, 104.8 (d, *J* = 3.4 Hz), 69.6, 66.7, 60.4, 47.1, 45.6, 45.6, 45.6, 40.2, 39.1, 35.3, 31.2, 30.2, 29.2, 8.2; HRMS (ESI) *m/z* calculated for C_31_H_34_FN_3_O_6_ [M + H^+^] 564.2504, found 564.2488.

##### 1-Cyclopropyl-6-fluoro-7-(4-(3-fluorobenzyloxy)-1-oxa-9-azaspiro[5.5]undec-9-yl)-4-oxo-1,4-dihydroquinoline-3-carboxylic Acid (**3p**)

Yield—58 mg (27%), brown solid, m.p. 78–80 °C. ^1^H-NMR (300 MHz, CDCl_3_) δ 15.01 (s, 1H), 8.73 (s, 1H), 7.97 (d, *J* = 13.1 Hz, 1H), 7.44 (d, *J* = 7.0 Hz, 1H), 7.35–7.27 (m, 1H), 7.13–7.03 (m, 2H), 7.03–6.92 (m, 1H), 4.56 (s, 2H), 3.93 (dt, *J* = 11.9, 4.5 Hz, 1H), 3.85–3.72 (m, 1H), 3.68–3.58 (m, 1H), 3.56–3.40 (m, 3H), 3.36–3.25 (m, 1H), 3.25–3.13 (m, 1H), 2.09 (br.d, *J* = 12.9 Hz, 2H), 2.03–1.95 (m, 1H), 1.95–1.90 (m, 1H), 1.90–1.86 (m, 1H), 1.85–1.74 (m, 1H), 1.72–1.64 (m, 1H), 1.64–1.57 (m, 1H), 1.42–1.34 (m, 2H), 1.24–1.15 (m, 2H); ^13^C-NMR (75 MHz, CDCl_3_) δ 177.1 (d, *J* = 2.6 Hz), 167.1, 163.1 (d, *J* = 245.9 Hz), 153.8 (d, *J* = 251.4 Hz), 147.4, 146.3 (d, *J* = 10.2 Hz), 141.4 (d, *J* = 7.1 Hz), 139.2, 130.1 (d, *J* = 8.2 Hz), 122.8 (d, *J* = 2.9 Hz), 119.6 (d, *J* = 7.9 Hz), 114.5 (d, *J* = 19.8 Hz), 114.2 (d, *J* = 20.3 Hz), 112.3 (d, *J* = 23.6 Hz), 108.1, 105.2 (d, *J* = 2.9 Hz), 71.6, 70.6, 69.3, 69.3, 58.9, 45.9, 45.9, 45.8, 45.7, 41.8, 37.1, 35.4, 32.5, 32.1, 8.3; HRMS (ESI) *m/z* calculated for C_29_H_30_F_2_N_2_O_5_ [M + H^+^] 525.2196, found 525.2212.

##### 1-Cyclopropyl-6-fluoro-4-oxo-7-(4-(pyridin-2-yloxy)-1-oxa-9-azaspiro[5.5]undec-9-yl)-1,4-dihydroquinoline-3-carboxylic Acid (**3q**)

Yield—110 mg (54%), white solid, m.p. 118–120 °C. ^1^H-NMR (300 MHz, CDCl_3_) δ 15.12 (s, 1H), 8.73 (s, 1H), 8.12 (d, *J* = 3.7 Hz, 1H), 7.95 (d, *J* = 13.1 Hz, 1H), 7.64–7.49 (m, 1H), 7.37 (d, *J* = 7.1 Hz, 1H), 6.94–6.79 (m, 1H), 6.70 (d, *J* = 8.3 Hz, 1H), 5.48–5.35 (m, 1H), 4.05–3.89 (m, 1H), 3.83–3.72 (m, 1H), 3.56–3.39 (m, 3H), 3.34–3.14 (m, 2H), 2.24 (d, *J* = 12.9 Hz, 1H), 2.15–1.99 (m, 3H), 1.91–1.76 (m, 3H), 1.73–1.68 (m, 1H), 1.43–1.32 (m, 2H), 1.25–1.14 (m, 2H); ^13^C-NMR (75 MHz, CDCl_3_) δ 177.0 (d, *J* = 2.6 Hz), 167.1, 162.9, 153.7 (d, *J* = 251.3 Hz), 147.2, 146.9, 146.4 (d, *J* = 10.3 Hz), 139.2, 138.8, 119.2 (d, *J* = 7.9 Hz), 116.8, 112.0 (d, *J* = 23.6 Hz), 111.7, 107.8, 104.9 (d, *J* = 3.4 Hz), 70.8, 67.5, 58.9, 45.8, 45.7, 45.6, 45.5, 41.2, 37.1, 35.4, 32.4, 31.9, 8.3; HRMS (ESI) calculated for C_27_H_28_FN_3_O_5_ [M + H^+^] 494.2086, found 494.2108.

##### 1-Cyclopropyl-6-fluoro-7-(4-methoxy-1-oxa-9-azaspiro[5.5]undec-9-yl)-4-oxo-1,4-dihydroquinoline-3-carboxylic Acid (**3r**)

Yield—84 mg (47%), white solid, m.p. 182–184 °C. ^1^H-NMR (300 MHz, CDCl_3_) δ 15.01 (s, 1H), 8.71 (s, 1H), 7.94 (d, *J* = 13.1 Hz, 1H), 7.41 (d, *J* = 6.7 Hz, 1H), 3.95–3.85 (m, 1H), 3.67–3.60 (m, 1H), 3.57–3.39 (m, 4H), 3.35 (s, 3H), 3.33–3.24 (m, 1H), 3.18 (t, *J* = 11.4 Hz, 1H), 2.14–1.93 (m, 3H), 1.91–1.81 (m, 2H), 1.75 (t, *J* = 11.7 Hz, 1H), 1.61–1.45 (m, 2H), 1.43–1.32 (m, 2H), 1.19 (br.s, 2H); ^13^C-NMR (75 MHz, CDCl_3_) δ 177.2 (d, *J* = 2.6 Hz), 167.2, 153.9 (d, *J* = 251.5 Hz), 147.5, 146.1 (d, *J* = 10.8 Hz), 139.2, 119.8 (d, *J* = 5.4 Hz), 112.4 (d, *J* = 23.5 Hz), 108.1, 105.4 (d, *J* = 2.1 Hz), 73.1, 70.6, 59.0, 55.7, 46.0, 45.9, 45.8, 41.4, 37.1, 35.5, 32.2, 31.8, 8.4; HRMS (ESI) *m/z* calculated for C_23_H_27_FN_2_O_5_ [M + H^+^] 431.1977, found 431.1995.

##### 1-Cyclopropyl-6-fluoro-4-oxo-7-(4-(pyrazin-2-yloxy)-1-oxa-9-azaspiro[5.5]undec-9-yl)-1,4-dihydroquinoline-3-carboxylic Acid (**3s**)

Yield—170 mg (83%), white solid, m.p. 128–130 °C. ^1^H-NMR (300 MHz, CDCl_3_) δ 15.08 (s, 1H), 8.72 (s, 1H), 8.18 (s, 1H), 8.11 (d, *J* = 2.4 Hz, 1H), 8.05 (br.s, 1H), 7.94 (d, *J* = 13.1 Hz, 1H), 7.37 (d, *J* = 6.8 Hz, 1H), 5.50–5.31 (m, 1H), 4.03–3.89 (m, 1H), 3.78 (t, *J* = 9.9 Hz, 1H), 3.58–3.41 (m, 3H), 3.29 (t, *J* = 11.1 Hz, 1H), 3.18 (t, *J* = 11.4 Hz, 1H), 2.24 (br.d, *J* = 14.7 Hz, 1H), 2.17–2.06 (m, 2H), 2.03 (br.s, 1H), 1.91–1.77 (m, 3H), 1.76–1.66 (m, 1H), 1.38 (br.d, *J* = 5.2 Hz, 2H), 1.19 (br.s, 2H); ^13^C-NMR (75 MHz, CDCl_3_) δ 176.8 (d, *J* = 0.8 Hz), 167.0, 159.4, 153.6 (d, *J* = 251.5 Hz), 147.2, 146.2 (d, *J* = 10.4 Hz), 140.5, 139.1, 136.6, 136.3, 119.1 (d, *J* = 7.9 Hz), 111.9 (d, *J* = 23.4 Hz), 107.9, 104.9 (d, *J* = 1.9 Hz), 70.8, 68.7, 58.7, 45.6, 45.5, 41.0, 37.1, 35.3, 32.2, 31.6, 8.2; HRMS (ESI) *m/z* calculated for C_26_H_27_FN_4_O_5_ [M + Na^+^] 517.1858, found 517.1881.

##### 1-Cyclopropyl-6-fluoro-7-(4-methyl-1-oxa-9-azaspiro[5.5]undec-9-yl)-4-oxo-1,4-dihydroquinoline-3-carboxylic Acid (**3t**)

Yield—113 mg (66%), yellow solid, m.p. 131–133 °C. ^1^H-NMR (300 MHz, CDCl_3_) δ 15.09 (s, 1H), 8.72 (s, 1H), 7.95 (d, *J* = 13.1 Hz, 1H), 7.36 (d, *J* = 7.1 Hz, 1H), 3.77 (dd, *J* = 12.1, 4.7 Hz, 1H), 3.61 (t, *J* = 12.2 Hz, 1H), 3.55–3.37 (m, 3H), 3.36–3.25 (m, 1H), 3.10 (t, *J* = 11.1 Hz, 1H), 2.37 (br.d, *J* = 13.9 Hz, 1H), 1.91–1.64 (m, 4H), 1.64–1.48 (m, 3H), 1.42–1.31 (m, 2H), 1.26–1.15 (m, 3H), 0.93 (d, *J* = 6.4 Hz, 3H); ^13^C-NMR (75 MHz, CDCl_3_) δ 176.8 (d, *J* = 2.0 Hz), 167.0, 153.6 (d, *J* = 251.2 Hz), 147.1, 146.4 (d, *J* = 10.3 Hz), 139.1, 119.0 (d, *J* = 8.0 Hz), 111.8 (d, *J* = 23.7 Hz), 107.8, 104.8 (d, *J* = 3.2 Hz), 69.7, 60.9, 45.7, 45.7, 45.6, 44.9, 39.2, 35.3, 34.7, 29.3, 25.1, 22.5, 8.2, 8.1; HRMS (ESI) *m/z* calculated for C_23_H_27_FN_2_O_4_ [M + Na^+^] 437.1847, found 437.1861.

##### 1-Cyclopropyl-6-fluoro-7-(4-hydroxy-1-oxa-9-azaspiro[5.5]undec-9-yl)-4-oxo-1,4-dihydroquinoline-3-carboxylic Acid (**3u**)

Yield—143 mg (83%), yellow solid, m.p. 250–252 °C. ^1^H-NMR (300 MHz, D_2_O) δ 8.43 (s, 1H), 7.68 (d, *J* = 13.6 Hz, 1H), 7.32 (d, *J* = 7.3 Hz, 1H), 4.08–3.93 (m, 1H), 3.86–3.75 (m, 1H), 3.62 (t, *J* = 11.5 Hz, 1H), 3.49–3.35 (m, 1H), 3.24–2.99 (m, 3H), 2.86 (t, *J* = 10.8 Hz, 1H), 2.06 (br.d, *J* = 14.2 Hz, 1H), 1.98–1.86 (m, 2H), 1.75 (br.s, 2H), 1.66–1.54 (m, 1H), 1.53–1.39 (m, 1H), 1.36–1.24 (m, 3H), 1.01 (br.s, 2H); ^13^C-NMR (75 MHz, D_2_O) δ 173.8 (d, *J* = 2.0 Hz), 170.8, 151.6 (d, *J* = 247.2 Hz), 145.5, 142.9 (d, *J* = 10.9 Hz), 136.9, 120.3 (d, *J* = 7.2 Hz), 115.1, 109.9 (d, *J* = 23.0 Hz), 104.6 (d, *J* = 1.9 Hz), 70.7, 62.1, 57.8, 44.4, 41.9, 36.1, 33.2, 32.8, 28.4, 6.0; HRMS (ESI) *m/z* calculated for C_22_H_25_FN_2_O_5_ [M + Na^+^] 439.1640, found 439.1652.

##### 1-Cyclopropyl-6-fluoro-4-oxo-7-(4-(4-trifluoromethylbenzyloxy)-1-oxa-9-azaspiro[5.5]undec-9-yl)-1,4-dihydroquinoline-3-carboxylic Acid (**3v**)

Yield—62 mg (26%), white solid, m.p. 86–88 °C. ^1^H-NMR (300 MHz, CDCl_3_) δ 15.00 (s, 1H), 8.74 (s, 1H), 7.99 (d, *J* = 13.1 Hz, 1H), 7.61 (d, *J* = 8.0 Hz, 2H), 7.46 (d, *J* = 7.8 Hz, 3H), 4.63 (s, 2H), 3.99–3.89 (m, 1H), 3.86–3.75 (m, 1H), 3.70–3.59 (m, 1H), 3.56–3.40 (m, 3H), 3.32 (t, *J* = 11.2 Hz, 1H), 3.20 (t, *J* = 11.4 Hz, 1H), 2.13–1.90 (m, 5H), 1.79–1.55 (m, 3H), 1.39 (d, *J* = 5.5 Hz, 2H), 1.20 (s, 2H); ^13^C-NMR (75 MHz, CDCl_3_) δ 177.2 (d, *J* = 2.2 Hz), 167.3, 153.9 (d, *J* = 251.5 Hz), 147.5, 146.2 (d, *J* = 6.0 Hz), 142.7, 139.2, 129.9 (q, *J* = 65.1, 32.7 Hz), 127.5, 125.5 (q, *J* = 3.7 Hz), 124.2 (q, *J* = 272.0 Hz), 119.8 (d, *J* = 5.5 Hz), 112.4 (d, *J* = 23.5 Hz), 108.2, 105.3 (d, *J* = 1.5 Hz), 71.8, 70.6, 69.3, 58.9, 45.9, 45.8, 41.8, 37.1, 35.5, 32.4, 32.1, 8.4; HRMS (ESI) *m/z* calculated for C_30_H_30_F_4_N_2_O_5_ [M + Na^+^] 597.1983, found 597.2006.

##### 1-Cyclopropyl-6-fluoro-7-(4-(4-fluorobenzyl)-1-oxa-9-azaspiro[5.5]undec-9-yl)-4-oxo-1,4-dihydroquinoline-3-carboxylic Acid (**3w**)

Yield—93 mg (44%), white solid, m.p. 87–89 °C. ^1^H-NMR (300 MHz, CDCl_3_) δ 15.05 (s, 1H), 8.74 (s, 1H), 7.96 (d, *J* = 12.9 Hz, 1H), 7.42 (br.s, 1H), 7.14–6.93 (m, 4H), 3.88–3.73 (m, 1H), 3.62–3.29 (m, 5H), 3.20–3.05 (m, 1H), 2.58–2.45 (m, 2H), 2.41–2.28 (m, 1H), 1.97–1.82 (m, 2H), 1.77–1.68 (m, 1H), 1.62–1.48 (m, 3H), 1.43–1.32 (m, 2H), 1.29–1.13 (m, 4H); ^13^C-NMR (75 MHz, CDCl_3_) δ 177.2 (d, *J* = 2.4 Hz), 167.2, 161.6 (d, *J* = 243.9 Hz), 153.9 (d, *J* = 251.5 Hz), 147.4, 146.3 (d, *J* = 11.3 Hz), 139.3, 135.5 (d, *J* = 3.2 Hz), 130.5 (d, *J* = 7.7 Hz), 119.7 (d, *J* = 7.8 Hz), 115.2 (d, *J* = 21.1 Hz), 112.4 (d, *J* = 23.7 Hz), 108.2, 105.2 (d, *J* = 1.7 Hz), 69.8, 60.9, 46.1, 46.0, 45.9, 45.9, 43.0, 43.0, 39.3, 35.5, 32.6, 29.4, 8.4, 8.4; HRMS (ESI) *m/z* calculated for C_29_H_30_F_2_N_2_O_4_ [M + Na^+^] 531.2066, found 531.2084.

##### 1-Cyclopropyl-6-fluoro-7-(4-(4-fluorobenzyloxy)-1-oxa-9-azaspiro[5.5]undec-9-yl)-4-oxo-1,4-dihydroquinoline-3-carboxylic Acid (**3x**)

Yield—88 mg (38%), white solid, m.p. 89–91 °C. ^1^H-NMR (300 MHz, CDCl_3_) δ 15.04 (s, 1H), 8.74 (s, 1H), 7.98 (d, *J* = 13.1 Hz, 1H), 7.42 (d, *J* = 6.1 Hz, 1H), 7.31 (dd, *J* = 8.3, 5.5 Hz, 2H), 7.03 (t, *J* = 8.6 Hz, 2H), 4.52 (s, 2H), 3.98–3.87 (m, 1H), 3.84–3.71 (m, 1H), 3.68–3.57 (m, 1H), 3.55–3.38 (m, 3H), 3.36–3.24 (m, 1H), 3.17 (t, *J* = 11.7 Hz, 1H), 2.13–2.02 (m, 2H), 1.99–1.82 (m, 3H), 1.80–1.72 (m, 1H), 1.71–1.63 (m, 1H), 1.60–1.53 (m, 2H), 1.43–1.34 (m, 2H), 1.20 (br.s, 2H); ^13^C-NMR (75 MHz, CDCl_3_) δ 177.2 (d, *J* = 2.5 Hz), 167.2, 162.4 (d, *J* = 245.6 Hz), 153.9 (d, *J* = 251.5 Hz), 147.5, 146.1 (d, *J* = 6.5 Hz), 139.2, 134.3 (d, *J* = 3.1 Hz), 129.3 (d, *J* = 8.1 Hz), 119.9 (d, *J* = 6.9 Hz), 115.4 (d, *J* = 21.4 Hz), 112.5 (d, *J* = 23.4 Hz), 108.2, 105.5 (d, *J* = 2.6 Hz), 71.3, 70.6, 69.4, 59.0, 45.9, 45.9, 41.8, 37.1, 35.5, 32.3, 32.2, 8.4; HRMS (ESI) *m/z* calculated for C_32_H_34_FN_3_O_5_ [M + Na^+^] 547.2020, found 547.2018.

##### 1-Cyclopropyl-7-(4-cyclopropylmethoxy-1-oxa-9-azaspiro[5.5]undec-9-yl)-6-fluoro-4-oxo-1,4-dihydroquinoline-3-carboxylic Acid (**3y**)

Yield—86 mg (44%), yellow solid, m.p. 86–88 °C. ^1^H-NMR (300 MHz, CDCl_3_) δ 15.05 (s, 1H), 8.72 (s, 1H), 7.94 (d, *J* = 13.1 Hz, 1H), 7.40 (d, *J* = 7.0 Hz, 1H), 3.89 (dt, *J* = 11.9, 4.2 Hz, 1H), 3.71–3.64 (m, 1H), 3.63–3.55 (m, 1H), 3.54–3.40 (m, 3H), 3.34–3.23 (m, 3H), 3.21–3.10 (m, 1H), 2.13 (d, *J* = 13.8 Hz, 1H), 2.05–1.93 (m, 2H), 1.91–1.83 (m, 2H), 1.77–1.67 (m, 1H), 1.64–1.53 (m, 1H), 1.53–1.45 (m, 1H), 1.42–1.34 (m, 2H), 1.22–1.15 (m, 2H), 1.12–0.97 (m, 1H), 0.66–0.48 (m, 2H), 0.29–0.14 (m, 2H); ^13^C-NMR (75 MHz, CDCl_3_) δ 176.8, (d, *J* = 2.4 Hz), 166.9, 153.6 (d, *J* = 251.4 Hz), 147.1, 146.2 (d, *J* = 10.3 Hz), 139.1, 119.0 (d, *J* = 7.9 Hz), 111.7 (d, *J* = 23.7 Hz), 107.7, 104.9 (d, *J* = 3.2 Hz), 72.6, 71.1, 70.6, 59.0, 45.7, 45.6, 45.5, 45.5, 41.8, 37.4, 35.4, 32.3, 31.9, 10.9, 8.1, 3.0; HRMS (ESI) *m/z* calculated for C_26_H_31_FN_2_O_5_ [M + H^+^] 471.2290, found 471.2310.

##### 1-Cyclopropyl-6-fluoro-4-oxo-7-(4-(3-pyridin-3-yl(1,2,4)oxadiazol-5-ylmethyl)-1-oxa-9-azaspiro[5.5]undec-9-yl)-1,4-dihydroquinoline-3-carboxylic Acid (**3z**)

Yield—180 mg (77%), beige solid, m.p. 238–240 °C. ^1^H-NMR (300 MHz, CDCl_3_) δ 15.07 (s, 1H), 9.34–9.23 (m, 1H), 8.73 (dd, *J* = 4.9, 1.7 Hz, 1H), 8.70 (s, 1H), 8.33 (dt, *J* = 8.0, 1.9 Hz, 1H), 7.92 (d, *J* = 13.1 Hz, 1H), 7.43 (ddd, *J* = 8.0, 4.9, 0.7 Hz, 1H), 7.35 (d, *J* = 7.2 Hz, 1H), 3.84 (dd, *J* = 11.9, 4.3 Hz, 1H), 3.74–3.62 (m, 1H), 3.56–3.38 (m, 3H), 3.31 (td, *J* = 11.6, 2.9 Hz, 1H), 3.18–3.05 (m, 1H), 2.91 (d, *J* = 7.0 Hz, 2H), 2.42 (br.d, *J* = 13.6 Hz, 2H), 1.94–1.57 (m, 5H), 1.52–1.31 (m, 4H), 1.22–1.14 (m, 2H); ^13^C-NMR (75 MHz, CDCl_3_) δ 178.7, 176.7 (d, *J* = 2.4 Hz), 166.8, 166.3, 153.5 (d, *J* = 249.7 Hz), 151.9, 148.4, 147.0, 146.1 (d, *J* = 10.3 Hz), 139.1, 134.6, 123.6, 123.1, 118.9 (d, *J* = 7.8 Hz), 111.7 (d, *J* = 23.6 Hz), 107.7, 104.8 (d, *J* = 3.3 Hz), 69.8, 60.3, 45.5, 45.5, 42.3, 39.0, 35.3, 33.6, 32.3, 29.3, 29.2, 8.1; HRMS (ESI) *m/z* calculated for C_30_H_30_FN_5_O_5_ [M + H^+^] 560.2304, found 560.2323.

##### 7-(4-((*N*-Benzyl-*N*-methylcarbamoyl)methyl)-1-oxa-9-azaspiro[5.5]undec-9-yl)-1-cyclopropyl-6-fluoro-4-oxo-1,4-dihydroquinoline-3-carboxylic Acid (**3aa**)

Yield—143 mg (61%), white solid, m.p. 97–99 °C. ^1^H-NMR (300 MHz, CDCl_3_) δ 15.07 (s, 1H), 8.73 (s, 1H), 7.97 (d, *J* = 13.2 Hz, 1H), 7.40–7.20 (m, 5H), 7.18–7.11 (m, 1H), 4.66–4.52 (m, 2H), 3.81–3.63 (m, 2H), 3.56–3.39 (m, 3H), 3.38–3.26 (m, 1H), 3.21–3.09 (m, 1H), 2.98–2.91 (2s, 3H), 2.47–2.35 (m, 2H), 2.32–2.22 (m, 2H), 1.87–1.62 (m, 6H), 1.37 (br.d, *J* = 5.4 Hz, 2H), 1.24–1.14 (m, 3H); ^13^C-NMR (75 MHz, CDCl_3_) δ 176.8, 171.8, 171.4, (d, *J* = 1.7 Hz), 153.6 (d, *J* = 251.8 Hz), 147.3, 146.2 (d, *J* = 4.7 Hz), 138.1, 137.4, 136.6, 129.0, 128.6, 128.0, 127.7, 127.4, 126.2, 119.2 (d, *J* = 2.7 Hz), 111.9 (d, *J* = 24.6 Hz), 104.7 (d, *J* = 2.7 Hz), 69.9, 69.9, 60.8, 60.7, 53.3, 50.8, 45.7, 45.7, 42.8, 42.7, 40.4, 40.1, 39.2, 35.3, 34.9, 34.1, 32.9, 32.8, 29.3, 27.5, 27.5, 8.2; HRMS (ESI) *m/z* calculated for C_32_H_36_FN_3_O_5_ [M + H^+^] 562.2712, found 562.2697.

##### 7-(4-((*N*-Benzyl-*N*-cyclopropyl)amino)-1-oxa-9-azaspiro[5.5]undec-9-yl)-1-cyclopropyl-6-fluoro-4-oxo-1,4-dihydroquinoline-3-carboxylic Acid (**3ab**)

Yield—66 mg (29%), brown solid, m.p. 241–243 °C. ^1^H-NMR (300 MHz, DMSO-*d_6_*) δ 15.25 (s, 1H), 8.64 (s, 1H), 7.87 (dd, *J* = 13.3, 3.0 Hz, 1H), 7.78–7.62 (m, 2H), 7.57 (t, *J* = 7.2 Hz, 1H), 7.46 (d, *J* = 1.8 Hz, 3H), 4.42 (br.s, 2H), 3.83 (br.s, 2H), 3.70–3.55 (m, 2H), 3.52–3.43 (m, 2H), 3.36–3.20 (m, 2H), 3.12–2.96 (m, 1H), 2.76–2.53 (m, 1H), 2.30–2.12 (m, 2H), 2.01–1.90 (m, 1H), 1.90–1.83 (m, 1H), 1.82–1.67 (m, 2H), 1.66–1.42 (m, 1H), 1.31 (br.s, 3H), 1.19 (br.s, 2H), 0.96–0.76 (m, 2H), 0.74–0.57 (m, 1H); ^13^C-NMR (75 MHz, DMSO-*d_6_*) δ 176.8 (d, *J* = 2.7 Hz), 166.3, 153.5 (d, *J* = 249.1 Hz), 148.3, 145.8 (d, *J* = 10.2 Hz), 139.8, 132.4, 132.2, 129.8, 129.0, 118.9 (d, *J* = 7.8 Hz), 111.4 (d, *J* = 23.4 Hz), 107.4, 106.7 (d, *J* = 3.5 Hz), 71.2, 59.5, 55.9, 55.3. 45.8, 45.8, 45.7, 45.7, 45.6, 36.3, 29.1, 8.1, 8.0; HRMS (ESI) *m/z* calculated for C_32_H_36_FN_3_O_4_ [M + H^+^] 546.2763, found 546.2771.

##### 1-Cyclopropyl-7-(4-(3-cyclopropyl[1,2,4]oxadiazol-5-ylmethyl)-1-oxa-9-azaspiro[5.5]undec-9-yl)-6-fluoro-4-oxo-1,4-dihydroquinoline-3-carboxylic Acid (**3ac**)

Yield—102 mg (47%), white solid, m.p. 106–108 °C. ^1^H-NMR (300 MHz, CDCl_3_) δ 15.07 (s, 1H), 8.73 (s, 1H), 7.96 (d, *J* = 13.1 Hz, 1H), 7.36 (d, *J* = 6.2 Hz, 1H), 3.81 (dd, *J* = 11.5, 3.9 Hz, 1H), 3.64 (t, *J* = 11.8 Hz, 1H), 3.57–3.38 (m, 3H), 3.31 (t, *J* = 10.6 Hz, 1H), 3.10 (t, *J* = 11.1 Hz, 1H), 2.74 (d, *J* = 6.7 Hz, 2H), 2.43–2.20 (m, 2H), 2.11–2.03 (m, 1H), 1.90–1.80 (m, 1H), 1.77–1.58 (m, 4H), 1.41–1.17 (m, 6H), 1.08–0.96 (m, 4H); ^13^C-NMR (75 MHz, CDCl_3_) δ 175.5, 175.0 (d, *J* = 2.6 Hz), 170.4, 165.1, 151.7 (d, *J* = 251.3 Hz), 145.3, 144.4 (d, *J* = 10.4 Hz), 137.1, 117.4 (d, *J* = 8.0 Hz), 110.1 (d, *J* = 23.6 Hz), 105.9, 102.8 (d, *J* = 3.4 Hz), 67.7, 58.3, 43.6, 43.5, 43.5, 40.4, 37.0, 33.3, 31.7, 30.2, 27.3, 27.1, 6.2, 6.2, 5.8, 4.8; HRMS (ESI) *m/z* calculated for C_28_H_31_FN_4_O_5_ [M + Na^+^] 545.2171, found 545.2187.

##### 1-Cyclopropyl-6-fluoro-7-(4-(3-(2-methoxyethyl)[1,2,4]oxadiazol-5-ylmethyl)-1-oxa-9-azaspiro[5.5]undec-9-yl)-4-oxo-1,4-dihydroquinoline-3-carboxylic Acid (**3ad**)

Yield—138 mg (61%), brown solid, m.p. 101–103 °C. ^1^H-NMR (300 MHz, CDCl_3_) δ 15.03 (s, 1H), 8.70 (s, 1H), 7.93 (d, *J* = 13.1 Hz, 1H), 7.39 (d, *J* = 6.5 Hz, 1H), 3.80–3.73 (m, 2H), 3.70–3.58 (m, 1H), 3.55–3.44 (m, 2H), 3.43–3.30 (m, 4H), 3.12 (t, *J* = 11.5 Hz, 1H), 2.99 (t, *J* = 6.3 Hz, 2H), 2.79 (d, *J* = 6.7 Hz, 2H), 2.44–2.24 (m, 2H), 2.15–1.79 (m, 3H), 1.77–1.54 (m, 4H), 1.40–1.15 (m, 6H); ^13^C-NMR (75 MHz, CDCl_3_) δ 178.0, 177.1 (d, *J* = 2.5 Hz), 168.4, 167.2, 153.8 (d, *J* = 251.4 Hz), 147.4, 146.2 (d, *J* = 10.2 Hz), 139.2, 119.6 (d, *J* = 7.8 Hz), 112.3 (d, *J* = 23.6 Hz), 108.1, 105.2 (d, *J* = 2.0 Hz), 69.8, 69.2, 60.5, 58.8, 45.8, 45.8, 45.7, 42.6, 39.1, 35.4, 33.8, 32.4, 29.4, 29.3, 26.9, 8.4; HRMS (ESI) *m/z* calculated for C_28_H_33_FN_4_O_6_ [M + H^+^] 541.2457, found 541.2472.

##### 1-Cyclopropyl-6-fluoro-4-oxo-7-(4-(4-phenylpiperazin-1-yl)-1-oxa-9-azaspiro[5.5]undec-9-yl)-1,4-dihydroquinoline-3-carboxylic Acid (**3ae**)

Yield—170 mg (73%), pale brown solid, m.p. 117–119 °C. ^1^H-NMR (300 MHz, DMSO-*d_6_*) δ 15.23 (s, 1H), 8.64 (s, 1H), 7.86 (d, *J* = 12.8 Hz, 1H), 7.56 (d, *J* = 5.2 Hz, 1H), 7.21 (br.s, 2H), 6.93 (d, *J* = 7.2 Hz, 2H), 6.77 (br.s, 1H), 3.87–3.75 (m, 2H), 3.69–3.55 (m, 2H), 3.47–3.39 (m, 3H), 3.33–3.27 (m, 2H), 3.19–3.11 (m, 4H), 2.80–2.67 (m, 4H), 2.35–2.24 (m, 1H), 1.91–1.76 (m, 3H), 1.71–1.57 (m, 2H), 1.36–1.27 (m, 3H), 1.23–1.15 (m, 2H); ^13^C-NMR (75 MHz, DMSO-*d_6_*) δ 176.4 (d, *J* = 2.4 Hz), 166.1, 153.1 (d, *J* = 249.5 Hz), 150.7, 148.0, 145.6 (d, *J* = 10.2 Hz), 139.3, 129.0, 119.2, 118.3 (d, *J* = 7.0 Hz), 115.5, 110.9 (d, *J* = 22.8 Hz), 106.7, 106.4 (d, *J* = 4.0 Hz), 76.8, 71.8, 70.4, 59.4, 56.3, 48.4, 47.9, 45.5, 45.4, 45.3, 43.7, 38.0, 35.9, 29.1, 28.2, 7.6; HRMS (ESI) *m/z* calculated for C_32_H_37_FN_4_O_4_ [M + H^+^] 561.2872, found 561.2859.

##### 7-(4-((*N*-Benzoyl-*N*-cyclopropyl)amino)-1-oxa-9-azaspiro[5.5]undec-9-yl)-1-cyclopropyl-6-fluoro-4-oxo-1,4-dihydroquinoline-3-carboxylic Acid (**3af**)

Yield—188 mg (81%), white solid, m.p. 136–138 °C. ^1^H-NMR (300 MHz, CDCl_3_) δ 15.09 (s, 1H), 8.75 (s, 1H), 7.98 (d, *J* = 13.0 Hz, 1H), 7.52–7.43 (m, 3H), 7.42–7.35 (m, 3H), 4.66 (t, *J* = 9.7 Hz, 1H), 3.93 (dd, *J* = 11.1, 3.6 Hz, 1H), 3.77 (t, *J* = 11.8 Hz, 1H), 3.57–3.32 (m, 4H), 3.19 (t, *J* = 10.9 Hz, 1H), 2.58 (br.s, 1H), 2.47 (br.d, *J* = 13.2 Hz, 1H), 2.06–1.76 (m, 7H), 1.39 (br.s, 2H), 1.25–1.16 (m, 2H), 0.68–0.55 (m, 2H), 0.46 (s, 2H); ^13^C-NMR (75 MHz, CDCl_3_) δ 177.2 (d, *J* = 2.4 Hz), 173.2, 167.2, 153.8 (d, *J* = 251.7 Hz), 147.5, 145.9 (d, *J* = 8.6 Hz), 139.2, 137.9, 129.7, 128.1, 127.4, 119.9 (d, *J* = 7.2 Hz), 112.5 (d, *J* = 23.5 Hz), 108.2, 105.5, 71.2, 60.9, 51.4, 46.1, 46.0, 45.9, 45.9, 40.7, 39.4, 35.5, 31.3, 29.2, 28.7, 10.1, 10.1, 8.4; HRMS (ESI) *m/z* calculated for C_32_H_34_FN_3_O_5_ [M + H^+^] 560.2555, found 560.2567.

##### 1-Cyclopropyl-6-fluoro-7-(4-morpholin-4-yl-1-oxa-9-azaspiro[5.5]undec-9-yl)-4-oxo-1,4-dihydroquinoline-3-carboxylic Acid (**3ag**)

Yield—91 mg (45%), yellow solid, m.p. 187–189 °C. ^1^H-NMR (300 MHz, CDCl_3_) δ 14.97 (s, 1H), 8.72 (s, 1H), 7.96 (d, *J* = 13.2 Hz, 1H), 7.36 (d, *J* = 7.2 Hz, 1H), 3.94–3.84 (m, 1H), 3.78–3.69 (m, 4H), 3.68–3.58 (m, 1H), 3.54–3.39 (m, 3H), 3.39–3.28 (m, 1H), 3.19–3.06 (m, 1H), 2.70–2.52 (m, 5H), 2.32 (br.d, *J* = 14.0 Hz, 1H), 1.95–1.75 (m, 4H), 1.70–1.50 (m, 2H), 1.47–1.33 (m, 3H), 1.23–1.15 (m, 2H); ^13^C-NMR (75 MHz, CDCl_3_) δ 177.0 (d, *J* = 2.7 Hz), 166.8, 153.7 (d, *J* = 251.1 Hz), 147.1, 146.3 (d, *J* = 10.4 Hz), 139.2, 119.3 (d, *J* = 7.9 Hz), 112.1 (d, *J* = 23.7 Hz), 108.1, 104.7 (d, *J* = 3.6 Hz), 70.5, 67.1, 60.2, 56.9, 49.6, 45.7, 45.7, 45.6, 45.6, 39.2, 39.1, 35.2, 30.0, 28.9, 8.1, 8.1; HRMS (ESI) *m/z* calculated for C_26_H_32_FN_3_O_5_ [M + H^+^] 486.2399, found 486.2420.

##### 1-Cyclopropyl-7-(4-(1-cyclopropyl-3-isopropylureido)-1-oxa-9-azaspiro[5.5]undec-9-yl)-6-fluoro-4-oxo-1,4-dihydroquinoline-3-carboxylic Acid (**3ah**)

Yield—79 mg (35%), white solid, m.p. 130–132 °C. ^1^H-NMR (300 MHz, CDCl_3_) δ 15.11 (s, 1H), 8.74 (s, 1H), 7.97 (d, *J* = 13.1 Hz, 1H), 7.35 (d, *J* = 7.1 Hz, 1H), 5.17 (d, *J* = 7.6 Hz, 1H), 4.48 (tt, *J* = 12.1, 3.3 Hz, 1H), 4.02–3.91 (m, 1H), 3.86 (dd, *J* = 12.3, 5.0 Hz, 1H), 3.71 (t, *J* = 11.4 Hz, 1H), 3.56–3.49 (m, 1H), 3.49–3.39 (m, 2H), 3.38–3.27 (m, 1H), 3.14 (t, *J* = 10.9 Hz, 1H), 2.43 (br.d, *J* = 14.2 Hz, 1H), 2.33–2.24 (m, 1H), 2.13–1.96 (m, 1H), 1.86–1.68 (m, 7H), 1.38 (d, *J* = 7.0 Hz, 2H), 1.17 (d, *J* = 6.5 Hz, 7H), 0.91–0.83 (m, 2H), 0.82–0.73 (m, 2H); ^13^C-NMR (75 MHz, CDCl_3_) δ 176.9 (d, *J* = 2.5 Hz), 167.1, 158.6, 153.6 (d, *J* = 251.4 Hz), 147.2, 146.3 (d, *J* = 10.3 Hz), 139.1, 119.1 (d, *J* = 7.9 Hz), 111.9 (d, *J* = 23.5 Hz), 107.8, 104.8 (d, *J* = 3.4 Hz), 71.2, 60.9, 50.2, 45.7, 45.6, 45.6, 45.5, 42.4, 41.2, 39.3, 35.3, 31.6, 29.2, 24.7, 23.5, 23.5, 8.8, 8.2; HRMS (ESI) *m/z* calculated for C_29_H_37_FN_4_O_5_ [M + Na^+^] 563.2640, found 563.2665.

##### 1-Cyclopropyl-7-(4-(1-cyclopropyl-3-ethylureido)-1-oxa-9-azaspiro[5.5]undec-9-yl)-6-fluoro-4-oxo-1,4-dihydroquinoline-3-carboxylic Acid (**3ai**)

Yield—180 mg (82%), white solid, m.p. 151–153 °C. ^1^H-NMR (300 MHz, CDCl_3_) δ 15.14 (s, 1H), 8.71 (s, 1H), 7.92 (d, *J* = 13.1 Hz, 1H), 7.35 (d, *J* = 7.2 Hz, 1H), 5.32 (t, *J* = 5.4 Hz, 1H), 4.47 (tt, *J* = 12.3, 3.5 Hz, 1H), 3.85 (dd, *J* = 11.8, 4.1 Hz, 1H), 3.77–3.64 (m, 1H), 3.56–3.48 (m, 1H), 3.48–3.37 (m, 2H), 3.37–3.21 (m, 3H), 3.19–3.06 (m, 1H), 2.42 (br.d, *J* = 14.1 Hz, 1H), 2.35–2.24 (m, 1H), 1.99–1.91 (m, 2H), 1.83–1.75 (m, 2H), 1.75–1.65 (m, 3H), 1.44–1.32 (m, 2H), 1.21–1.16 (m, 2H), 1.14 (t, *J* = 7.2 Hz, 3H), 0.91–0.83 (m, 2H), 0.82–0.73 (m, 2H); ^13^C-NMR (75 MHz, CDCl_3_) δ 177.2 (d, *J* = 2.7 Hz), 167.4, 159.4, 153.8 (d, *J* = 251.3 Hz), 147.4, 146.5 (d, *J* = 10.4 Hz), 139.3, 119.5 (d, *J* = 7.9 Hz), 112.3 (d, *J* = 23.5 Hz), 108.1, 104.9 (d, *J* = 3.6 Hz), 71.4, 61.1, 50.5, 45.9, 45.8, 45.7, 45.7, 41.3, 39.5, 35.6, 35.4, 31.7, 29.3, 25.0, 15.7, 8.8, 8.3; HRMS (ESI) *m/z* calculated for C_28_H_35_FN_4_O_5_ [M + Na^+^] 549.2484, found 549.2502.

##### 7-(4-(*N*-Acetyl-*N*-cyclopropylamino)-1-oxa-9-azaspiro[5.5]undec-9-yl)-1-cyclopropyl-6-fluoro-4-oxo-1,4-dihydroquinoline-3-carboxylic Acid (**3aj**)

Yield—80 mg (39%), beige solid, m.p. 120–122 °C. ^1^H-NMR (300 MHz, CDCl_3_) δ 15.07 (s, 1H), 8.74 (s, 1H), 7.96 (d, *J* = 13.1 Hz, 1H), 7.44 (d, *J* = 6.8 Hz, 1H), 4.55 (t, *J* = 11.9 Hz, 1H), 3.88 (dd, *J* = 11.7, 4.5 Hz, 1H), 3.73 (t, *J* = 11.8 Hz, 1H), 3.59–3.33 (m, 4H), 3.18 (t, *J* = 11.1 Hz, 1H), 2.58–2.41 (m, 2H), 2.23 (s, 3H), 2.16–2.03 (m, 1H), 1.97–1.85 (m, 2H), 1.84–1.67 (m, 4H), 1.41 (d, *J* = 6.6 Hz, 2H), 1.26–1.16 (m, 2H), 1.01–0.90 (m, 2H), 0.89–0.79 (m, 2H); ^13^C-NMR (75 MHz, CDCl_3_) δ 177.2 (d, *J* = 2.7 Hz), 174.1, 167.1, 153.9 (d, *J* = 251.4 Hz), 147.5, 146.0 (d, *J* = 10.5 Hz), 139.3, 119.9 (d, *J* = 8.0 Hz), 112.5 (d, *J* = 23.6 Hz), 108.3, 105.3 (d, *J* = 2.3 Hz), 71.2, 61.0, 50.7, 46.1, 46.0, 46.0, 45.9, 40.7, 39.4, 35.5, 31.3, 29.3, 28.3, 23.8, 9.5, 9.4, 8.4; HRMS (ESI) *m/z* calculated for C_27_H_32_FN_3_O_5_ [M + Na^+^] 520.2218, found 520.2237.

### 3.2. Bacterial Susceptibility Testing

Testing was performed for the following microorganisms: *Staphylococcus aureus* (ATCC 25923), *Klebsiella pneumoniae* (1062^®^), *Acinetobacter baumannii* (987^®^), *Pseudomonas aeruginosa* (7292/5^®^) and *Bacillus cereus* (138^®^) for compounds **3a–aj** as well as ciprofloxacin (positive control) using the conventional Kirby–Bauer disk diffusion test [22] under the Standard Operating Procedure of The European Committee on Antimicrobial Susceptibility Testing (EUCAST) [23]. Disks containing 5 mg of ciprofloxacin were used. Solutions of compounds **1a**–**s**, **2a**–**s** and **4** in dimethyl sulfoxide (1 mg/10 mL) were prepared and diluted to a volume of 1 mL with deionized water. The resulting solution’s aliquots (5 mL) were added to a Petri dish containing Mueller–Hinton agar inoculated with a bacterial suspension (McFarland OD ¼ 0.5). After the drying of the compound solution, the Petri dish was incubated at 37 °C for 18 h. By measuring the bacterial growth inhibition zone diameter around the disc with ciprofloxacin or the compounds’ dried solution circular spot, the susceptibility to a drug was assessed. Additionally, minimum inhibitory concentrations (MIC, µg/mL) were determined using serial broth dilutions [24].

## 4. Conclusions

In summary, we explored the possibility of using spirocyclic piperidines (amenable to the Prins cyclization of protected 4-piperidone and homoallylic alcohol in aqueous mineral acid and subsequent functional group interconversions) in the design of ciprofloxacin analogs. Using the literature-established procedure of activating the halogen-substituted fluoroquinolone core by boron complexation, 36 new ciprofloxacin analogs were synthesized and tested against two gram-positive and three gram-negative bacterial strains. The activity profile of the new spirocyclic compounds displayed significant sensitivity to the peripheral groups in the 1-oxa-9-azaspiro[5.5]undecane moiety. Overall, the new set of derivatives was distinctly active against two of the five strains: gram-negative *Acinetobacter baumannii* 987^®^ and gram-positive *Bacillus cereus* 138^®^. Towards these two strains, a large group of compounds displayed equal or higher potency than ciprofloxacin. These findings substantially expand the utility of spirocyclic motifs in medicinal chemistry design and further attest to the privileged character of spirocycles.

## Figures and Tables

**Table 1:** Antibacterial activity (MIC, mg/mL) of compounds **3a**–**aj** against five bacterial strains in comparison with ciprofloxacin (-: no activity; +: active but MIC is higher than that of ciprofloxacin; ++: MIC same as that of ciprofloxacin; +++: MIC lower than that of ciprofloxacin; NT: not tested).

Compound	Yield, %	*Staphylococcus Aureus* ATCC 25923	*Klebsiella pneumoniae* 1062^®^	*Acinetobacter baumannii* 987^®^	*Pseudomonas aeruginosa* 7292/5^®^	*Bacillus cereus* 138^®^
**3a**	44	-	-	-	-	NT
**3b**	22	+	+	+++ *^a^*	-	+
**3c**	27	+	-	-	-	NT
**3d**	59	+	+	++ *^b^*	+	+
**3e**	50	+	-	+++ *^a^*	-	NT
**3f**	59	+	+	++ *^b^*	-	++ *^b^*
**3g**	86	+	+	+++ *^a^*	-	NT
**3h**	22	+	-	-	-	NT
**3i**	63	+	+	+	-	NT
**3j**	29	+	+	++ *^b^*	-	NT
**3k**	39	+	-	++ *^b^*	-	NT
**3l**	25	+	-	+++ *^a^*	-	++ *^b^*
**3m**	51	+	-	-	-	NT
**3n**	35	+	+	+++ *^a^*	-	++ *^b^*
**3o**	93	+	-	+++ *^a^*	-	NT
**3p**	27	+	-	+++ *^a^*	-	NT
**3q**	54	+	-	++ *^b^*	-	NT
**3r**	47	+	-	++ *^b^*	+	++ *^b^*
**3s**	83	+	+	-	-	NT
**3t**	66	+	-	+	-	NT
**3u**	83	+	+	++ *^b^*	-	++ *^b^*
**3v**	26	+	-	+++ *^a^*	-	NT
**3w**	44	-	-	+++ *^a^*	-	NT
**3x**	38	+	-	+	-	NT
**3y**	44	+	+	-	-	NT
**3z**	77	+	-	+++ *^a^*	-	NT
**3aa**	61	+	-	+++ *^a^*	-	NT
**3ab**	29	+	-	+++ *^a^*	-	NT
**3ac**	47	+	+	+	-	++ *^b^*
**3ad**	61	+	+	+++ *^a^*	-	++ *^b^*
**3ae**	73	+	-	++ *^b^*	-	NT
**3af**	81	+	-	+++ *^a^*	-	NT
**3ag**	45	+	+	+	-	++ *^b^*
**3ah**	35	+	-	-	-	NT
**3ai**	80	+	-	-	-	NT
**3aj**	39	+	+	-	-	NT
Ciprofloxacin (MIC)		0.3 μg/mL	0.3 μg/mL	0.3 μg/mL	0.15 μg/mL	0.3 μg/mL

*^a^* MIC = 0.15 μg/mL; *^b^* MIC = 0.3 μg/mL.

## Data Availability

Data available from the corresponding authors upon reasonable request.

## Sample Availability

Samples of the compounds are available from the authors upon reasonable request.
